# A post-ingestive amino acid sensor promotes food consumption in *Drosophila*

**DOI:** 10.1038/s41422-018-0084-9

**Published:** 2018-09-12

**Authors:** Zhe Yang, Rui Huang, Xin Fu, Gaohang Wang, Wei Qi, Decai Mao, Zhaomei Shi, Wei L. Shen, Liming Wang

**Affiliations:** 1 Life Sciences Institute, Zhejiang University, Hangzhou, 310058 Zhejiang China; 2 Innovation Center for Cell Signaling Network, Zhejiang University, Hangzhou, 310058 Zhejiang China; 3 Key Laboratory for Biorheological Science and Technology of Ministry of Education, State and Local Joint Engineering Laboratory for Vascular Implants, Bioengineering College, Chongqing University, Chongqing, 400030 China; 4 Medical School, Chongqing University, 400030 China; 5 School of Life Science and Technology, ShanghaiTech University, Shanghai, 201210 China; 6 Institute of Neuroscience, Shanghai Institutes for Biological Sciences, Chinese Academy of Sciences, Shanghai, 200031 China; 7 University of Chinese Academy of Sciences, Beijing, 100049 China; 8 Gene Regulatory Laboratory, School of Medicine, Tsinghua University, Beijing, 100084 China

## Abstract

Adequate protein intake is crucial for the survival and well-being of animals. How animals assess prospective protein sources and ensure dietary amino acid intake plays a critical role in protein homeostasis. By using a quantitative feeding assay, we show that three amino acids, L-glutamate (L-Glu), L-alanine (L-Ala) and L-aspartate (L-Asp), but not their D-enantiomers or the other 17 natural L-amino acids combined, rapidly promote food consumption in the fruit fly *Drosophila melanogaster*. This feeding-promoting effect of dietary amino acids is independent of mating experience and internal nutritional status*.* In vivo and ex vivo calcium imagings show that six brain neurons expressing diuretic hormone 44 (DH44) can be rapidly and directly activated by these amino acids, suggesting that these neurons are an amino acid sensor. Genetic inactivation of DH44^+^ neurons abolishes the increase in food consumption induced by dietary amino acids, whereas genetic activation of these neurons is sufficient to promote feeding, suggesting that DH44^+^ neurons mediate the effect of dietary amino acids to promote food consumption. Single-cell transcriptome analysis and immunostaining reveal that a putative amino acid transporter, CG13248, is enriched in DH44^+^ neurons. Knocking down CG13248 expression in DH44^+^ neurons blocks the increase in food consumption and eliminates calcium responses induced by dietary amino acids. Therefore, these data identify DH44^+^ neuron as a key sensor to detect amino acids and to enhance food intake via a putative transporter CG13248. These results shed critical light on the regulation of protein homeostasis at organismal levels by the nervous system.

## Introduction

Proteins are the most abundant macromolecules in living organisms with a vast array of biological functions. Adequate and balanced protein intake is therefore vital for the survival, reproduction, and well-being of animals. In fruit flies *Drosophila melanogaster*, several layers of regulatory mechanisms are involved in the regulation of protein intake.

First, prolonged protein deprivation leads to feeding preference towards protein-rich diet and increased protein consumption in both larvae and adults.^1–6^ In the adult brain, the detection of protein hunger and the induction of subsequent protein feeding involve the dynamic re-organization of a small group of dopaminergic neurons.^1^

Second, shortly after feeding on protein-rich diet, the insulin-producing cells (IPCs) in the fly brain are activated and exert robust suppressive effect on protein feeding in both larvae^7^ and adults.^8^ In larvae, the activation of IPCs after protein intake can be directly triggered by circulating L-leucine (L-Leu) via a leucine transporter minidiscs (MND) and glutamate dehydrogenase (GDH).^9^ In adults, IPCs can be activated by a fat body-derived satiety hormone named female-specific independent of transformer (FIT).^10^

Last but not least, amino acid composition in food also modulates flies’ food intake behaviors. Larval flies detect and reject food sources lacking one or more essential amino acids (essential amino acid deficiency, EAAD), which is regulated by a subset of dopaminergic neurons.^11^ In adult flies, EAAD induces potent feeding preference towards protein-rich food, and the commensal bacteria *Acetobacter pomorum* and *Lactobacilli* are important modulators of this behavioral shift.^12^ Collectively, these neural mechanisms ensure fruit flies to assess their internal amino acid adequacy and to secure adequate and balanced intake of amino acids.

Meanwhile, fruit flies must be able to detect the quality and quantity of dietary amino acids in potential food sources and modulate food consumption accordingly. In mammals, dietary amino acids elicit umami taste via the T1R1/T1R3 taste receptor located on the oral taste buds, which is believed to play a fundamental role in facilitating the evaluation and consumption of potential protein sources.^13^ Fruit flies, however, lack the homolog of mammalian umami taste receptor and must employ distinct amino acid sensing mechanisms.^4,6,14^ Ir76b, an inotropic chemosensory receptor, has been shown to mediate amino acid sensing in both larvae and adults. In larval flies, Ir76b is required for behavioral attraction to amino acids.^15^ In adults, Ir76b is expressed in tarsal, labella, and pharyngeal taste neurons.^16,17^ Ir76b, and the taste neurons expressing Ir76b, have been suggested to directly sense dietary amino acids and modulate their effect on food preference and consumption.^16,18^ Amino acid sensing via Ir76b^+^ neurons is dependent on the internal nutritional state of flies.^16,18^

In this study, we examined the effect of dietary amino acids to modulate food intake in adult fruit flies and sought to identify additional amino acid sensor. We found that dietary amino acids significantly promoted food intake independent of Ir76b signaling as well as flies’ internal nutritional status. Among all 20 natural amino acids, only three of them, L-Glu, L-Ala and L-Asp, but not their unnatural D-enantiomers, enhanced food consumption. Recording the calcium transients in vivo and ex vivo in the fly brain revealed that these three amino acids rapidly and directly activated a small group of neurons expressing diuretic hormone 44 (DH44), the homolog of mammalian corticotropin-releasing hormone (CRH). Genetic silencing and activation of these DH44^+^ neurons showed that they were both necessary and sufficient for dietary amino acids and yeast extract to promote food consumption.

We further investigated the molecular mechanism underlying the activation of DH44^+^ neurons by specific dietary amino acids. By single-cell transcriptome analysis, we identified that CG13248, a putative amino acid transporter, was highly expressed in DH44^+^ neurons and required for dietary amino acids to promote feeding. Furthermore, knocking out two DH44 receptors and DH44 itself completely abolished the increase in food consumption by dietary amino acids, as genetically silencing DH44^+^ neurons, suggesting that these receptors acted in the same circuitry to regulate amino acid consumption.

In aggregate, we have identified DH44^+^ neurons as a novel sensor in the fly brain that rapidly detects specific dietary amino acids and promotes amino acid consumption. These dietary amino acids may enter DH44^+^ neurons via CG13248 to excite these neurons, which may release DH44 neuropeptide and promote feeding through two DH44 receptors in downstream neurons.

## Results

### Dietary amino acids rapidly promote food consumption

Previous studies have shown that dietary amino acids modulate multiple aspects of flies’ food intake behavior, including food preference and food consumption.^1–6,10,11^ Peripherally, an ionotropic receptor Ir76b is sensitive to a number of amino acids in food and modulate food preference in both larvae and adults.^15,16,18^ We aimed to further examine the effect of dietary amino acid on flies’ food intake behaviors and the presence of additional amino acid sensor(s).

**Fig. 1:**
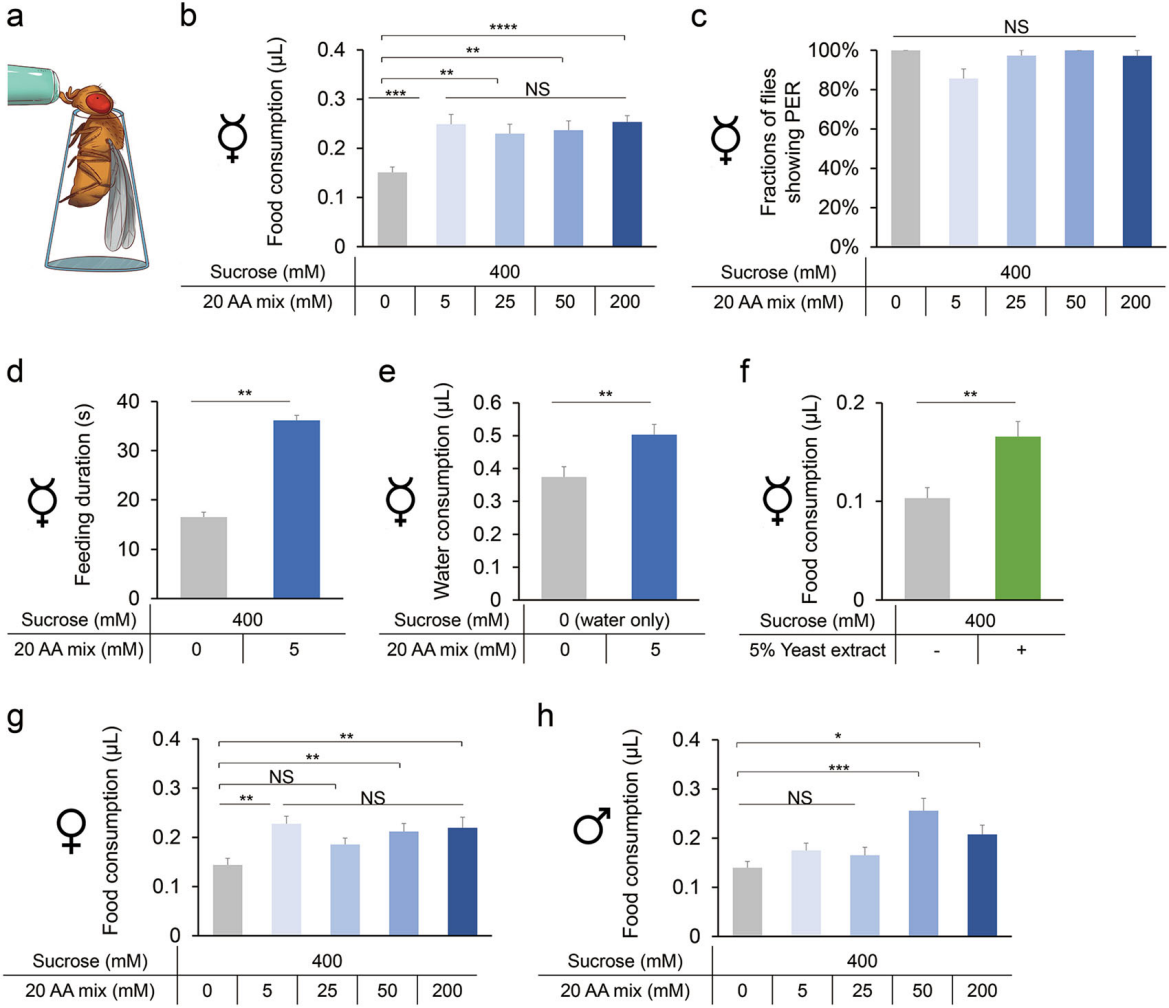
Promotion of food consumption by dietary amino acids. **a** Schematic illustration of the MAFE assay. **b** Volume of 400 mM sucrose (grey) or 400 mM sucrose plus different concentrations of amino acid mixture (blue) consumed by *Canton-S* virgin females fed *ad libitum* (*n* = 21–59). The composition of the amino acid mixture is shown in Supplementary information, Table S1.^20^ **c** Fractions of *Canton-S* virgin females showing PER responses to sucrose alone (grey) or sucrose plus different concentrations of amino acid mixture (blue) (*n* = 38–40). **d** Feeding duration of *Canton-S* virgin flies when fed with sucrose alone (grey) or sucrose plus amino acid mixture (blue) (*n* = 20). **e** Volume of water consumption in the presence or absence of 5 mM amino acid mixture by water-deprived *Canton-S* virgin females (*n* = 28–32). **f** Volume of sucrose solution consumed by *Canton-S* virgin females, in the presence or absence of 5% yeast extract (*n* = 17–18). **g, h** Volume of food consumed by mated *Canton-S* females (**g**) or males (**h**) fed *ad libitum* (*n* = 18–24). Symbols indicate the type of flies used in each experiment (virgin females, mated females, or males). Data are shown as means ± SEM. NS, *P* > 0.05; *P < 0.05; **P < 0.01; ***P < 0.001; ****P < 0.0001

To this end, we first examined the effect of adding varying concentrations of amino acids on sucrose consumption. By using the MAFE (Manual Feeding) assay that measured the volume of ingested food of individual flies (Fig. 1a),^19^ we found that the addition of amino acids significantly increased sucrose consumption of virgin female flies fed ad libitum (Fig. 1b). Addition of 5 mM of amino acid mixture increased food consumption by ~50%, which was close to a saturated level since further increase of amino acid concentration did not induce more sucrose consumption (Fig. 1b). Notably, amino acids alone, or the addition of amino acids to sweet and bitter solutions, did not alter the proboscis extension reflex (PER) responses in these fed flies, the initial step of food consumption behavior (Fig. 1c and Supplementary information, Fig. S1), suggesting that the increase in sucrose consumption was likely due to increased feeding duration in the presence of dietary amino acids (Fig. 1d). Contradictory to our results, a previous report showed that several amino acids, especially L-Cysteine (L-Cys) and L-Phenylalanine (L-Phe), could induce PER responses when applied to the labellum of pre-starved flies.^4^ As suggested by a more recent report, a plausible explanation is that the internal nutritional status may influence peripheral amino acid responses.^18^

To test whether amino acids merely enhanced feeding towards sucrose or they imposed a general stimulating effect of food consumption, we examined water consumption in water-deprived flies. These water-deprived flies consumed significantly more water in the presence of amino acids (Fig. 1e), indicating that dietary amino acids alone are capable of promoting feeding behavior. Yeast is the major protein source for fruit flies in their natural habitats and in laboratory conditions.^20,21^ We also found that adding 5% yeast extract to sucrose solution significantly promoted food consumption (Fig. 1f). Since intact protein macromolecules did not enhance food consumption (Supplementary information, Fig. S2), it is likely that the free amino acids in yeast extract, rather than the peptides or whole proteins, rapidly promote general food consumption of fruit flies.

As previously shown, female flies’ mating experience significantly enhanced their preference towards yeast, which may be due to their increased protein requirement for egg production.^2,3^ Nevertheless, we found that dietary amino acids enhanced feeding to similar extents in both virgin (Fig. 1b) and mated females (Fig. 1g). Dietary amino acids also enhanced feeding in male flies, although to a lesser extent (Fig. 1h).

Feeding preference towards protein-rich diet is dependent on, or at least greatly enhanced, by previous protein deprivation of fruit flies.^1–5,10,16,18,22^ However, flies deprived of protein sources exhibited comparable amino acid consumption to those previously supplied with yeast extract (Supplementary information, Fig. S3), suggesting that dietary amino acids enhance feeding independent of flies’ internal nutritional status. This seemingly discrepancy may also reflect the differences between the MAFE assay and other food intake related assays (see Discussions).

We also asked whether enhanced food consumption by dietary amino acids was only seen in immobilized flies in the MAFE assay, or rather reflected a more general amino acid sensing mechanism. By using the FLIC (Fly Liquid-Food Interaction Counter) assay,^23^ we found that individual free-moving flies also exhibited longer duration of food contact in the presence of amino acids than that of sucrose only, confirming a more general effect of dietary amino acids to promote food consumption (Supplementary information, Fig. S4a).

### L-Glu, L-Ala and L-Asp are sufficient to promote food consumption

**Fig. 2:**
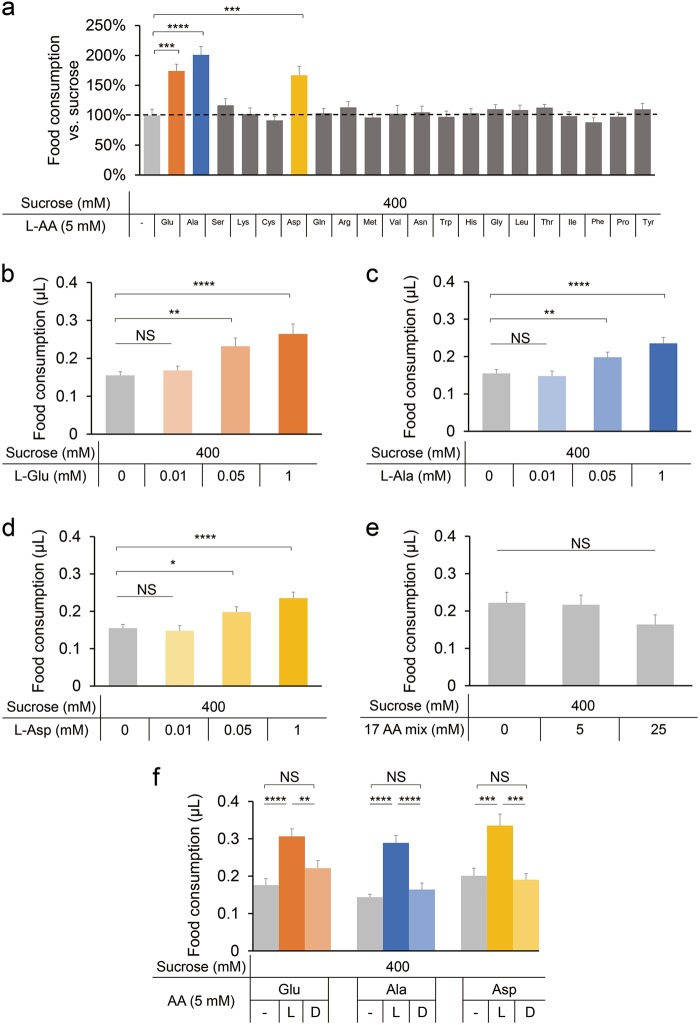
Promotion of food consumption by L-Glu, L-Ala and L-Asp. **a** Changes in food consumption by the addition of 5 mM amino acid to 400 mM sucrose by *Canton-S* flies (*n* = 20–26) (except tyrosine, for which we used its saturated concentration of ~2.7 mM). **b–d** Volume of 400 mM sucrose plus different concentrations of L-Glu (**b**), L-Ala (**c**), and L-Asp (**d**) consumed by *Canton-S* flies (*n* = 15–37). **e** Volume of 400 mM sucrose plus different concentrations of the other 17 amino acids combined consumed by *Canton-S* flies (*n* = 13–15). **f** Volume of 400 mM sucrose or 400 mM sucrose plus 5 mM L- or D-amino acid consumed by *Canton-S* flies (*n* = 20–26). Virgin females were used for all experiments shown in this figure. Data are shown as means ± SEM. NS, *P* > 0.05; *P < 0.05; **P < 0.01; ***P < 0.001; ****P < 0.0001

Since dietary amino acid mixture promoted food consumption, we sought to identify the specific amino acid(s) that mediated such effect in fruit flies. We found that out of 20 natural L-amino acids, only three of them, L-Glu, L-Ala and L-Asp, significantly enhanced food consumption (Fig. 2a).

All three amino acids promoted food consumption in a dose-dependent manner, starting from a concentration of 0.05 mM (Fig. 2b–d). In contrast, the mixture of the 17 other amino acids did not promote food consumption, even at much higher concentrations (Figs. 2e, 5 and 25 mM). In mammals, only the L-amino acids, but not their D-enantiomers, enhance food consumption.^13,24^ Similar to mammalian taste, we found that D-Glu, D-Ala and D-Asp could not promote food consumption (Fig. 2f).

### DH44 and DH44 receptors are required for dietary amino acids to promote food consumption

Dietary amino acids enhanced food consumption without altering the peripheral PER responses (Fig. 1b and Supplementary information, Fig. S1). In addition, we found that Ir76b, an amino acid sensor expressed in tarsal, labella and pharyngeal taste neurons,^15–17^ was not required for enhanced food consumption by dietary amino acids in the MAFE assay (Supplementary information, Fig. S5). *Ir761*^*1/1*^ and *Ir76b*^*1/+*^ flies even exhibited a modest but significant increase in food consumption compared to controls in the presence of dietary amino acids (Supplementary information, Fig. S5). Notably, Ir76b^+^ neurons seem to be required for feeding preference to yeast in free-moving flies,^16,18^ which again highlights the potential difference between the MAFE assay and other feeding assays employing free-moving flies.

**Fig. 3:**
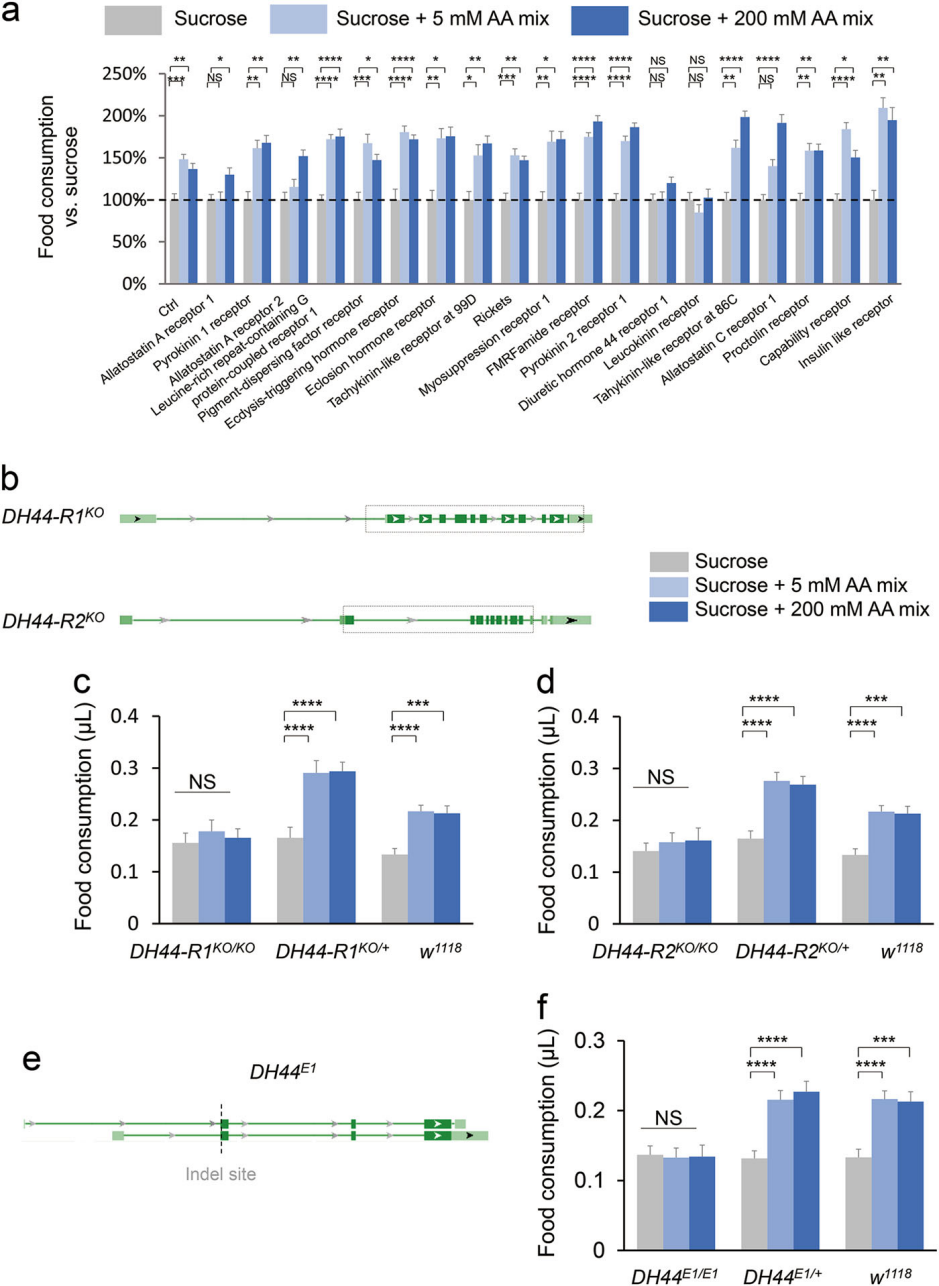
Requirement of DH44 signaling for the increase in food consumption by dietary amino acids. **a** Changes in food consumption by the addition of different concentrations of amino acid mixture to 400 mM sucrose consumed by flies with pan-neuronal RNAi knockdown of the indicated neuropeptide receptors (*n* = 19–35). Food consumption of 400 mM sucrose alone is used as the baseline (grey, dotted line). **b** Genomic structure of *DH44-R1* and *DH44-R2* genes. Dotted boxes outline the deleted DNA fragments by CRISPR/Cas9. **c, d** Volume of 400 mM sucrose (grey) or 400 mM sucrose plus different concentrations of amino acid mixture (blue) consumed by flies with the indicated genotypes (*n* = 19–28). **e** Genomic structure of *DH44* genes. Dotted line outlines the indel site induced by CRISPR/Cas9. **f** Volumes of 400 mM sucrose (grey) or 400 mM sucrose plus different concentrations of amino acid mixture (blue) consumed by flies with the indicated genotypes (*n* = 19–28). Virgin females were used for all experiments shown in this figure. Data are shown as means ± SEM. NS, *P* > 0.05; *P < 0.05; **P < 0.01; ***P < 0.001; ****P < 0.0001

Nevertheless, we suspected that an additional amino acid sensor in the central nervous system might mediate the effect of dietary amino acids to promote food consumption in the MAFE assay. To identify the putative amino acid sensor, we screened a collection of candidate neuropeptide receptors by using the MAFE assay (Fig. 3a). Among the receptors we screened, knocking down DH44 Receptor 1 (DH44-R1) and leucokinin receptor (LKR) in the nervous system by using a pan-neuronal *elav-GAL4* driver abolished the effect of dietary amino acids to enhance food consumption. In relation to our findings, LKR signaling has been shown to regulate different aspects of feeding behavior, including the meal size and feeding frequency^25^ and sleep regulation after protein intake.^26^ In this study, we focused on the function of DH44 signaling in amino acid sensing and food consumption.

DH44 is the fly homolog of mammalian CRH.^27^ Like mammalian CRH, DH44 has two receptors, DH44-R1 and DH44-R2 (Fig. 3b).^28^ DH44-R1 is expressed in the nervous system and is involved in feeding control, whereas DH44-R2 is expressed in gut enteroendocrine cells and regulates gut motility and excretion.^29^ We generated genetic mutants for both DH44 receptors, *DH44-R1* and *DH44-R2*, by CRISPR/Cas9-mediated gene editing (Fig. 3b). Both *DH44-R1*^*KO*^ and *DH44-R2*^*KO*^ mutants exhibited completely abolished responses to dietary amino acids (Fig. 3c, d).

These results suggest that DH44 signaling is required for dietary amino acids to promote food consumption. We thus generated a genetic mutant for *DH44 (DH44*^*E1/E1*^*)* by CRISPR/Cas9-mediated gene editing (Fig. 3e) and confirmed that *DH44* was required for the increase in food consumption by dietary amino acids (Fig. 3f). In the FLIC assay, free-moving *DH44*^*E1/E1*^ mutants also exhibited no increase in duration of food contact in the presence of amino acids as well as a general reduction in food intake (Supplementary information, Fig. S4b). Collectively, DH44 signaling is required for the effect of dietary amino acids to promote food consumption. Notably, immobilized *DH44*^*E1/E1*^ mutants did not exhibit a general reduction in food intake in the MAFE assay (Fig. 3f and Supplementary information, Fig. S4c), again suggesting a potential discrepancy between these two feeding assays. Specifically, DH44 signaling may be required for free-moving flies to search, locate, and ingest food in the FLIC assays, but not for immobilized flies in the MAFE assays.

### DH44^+^ neurons are rapidly activated during amino acid feeding

**Fig. 4:**
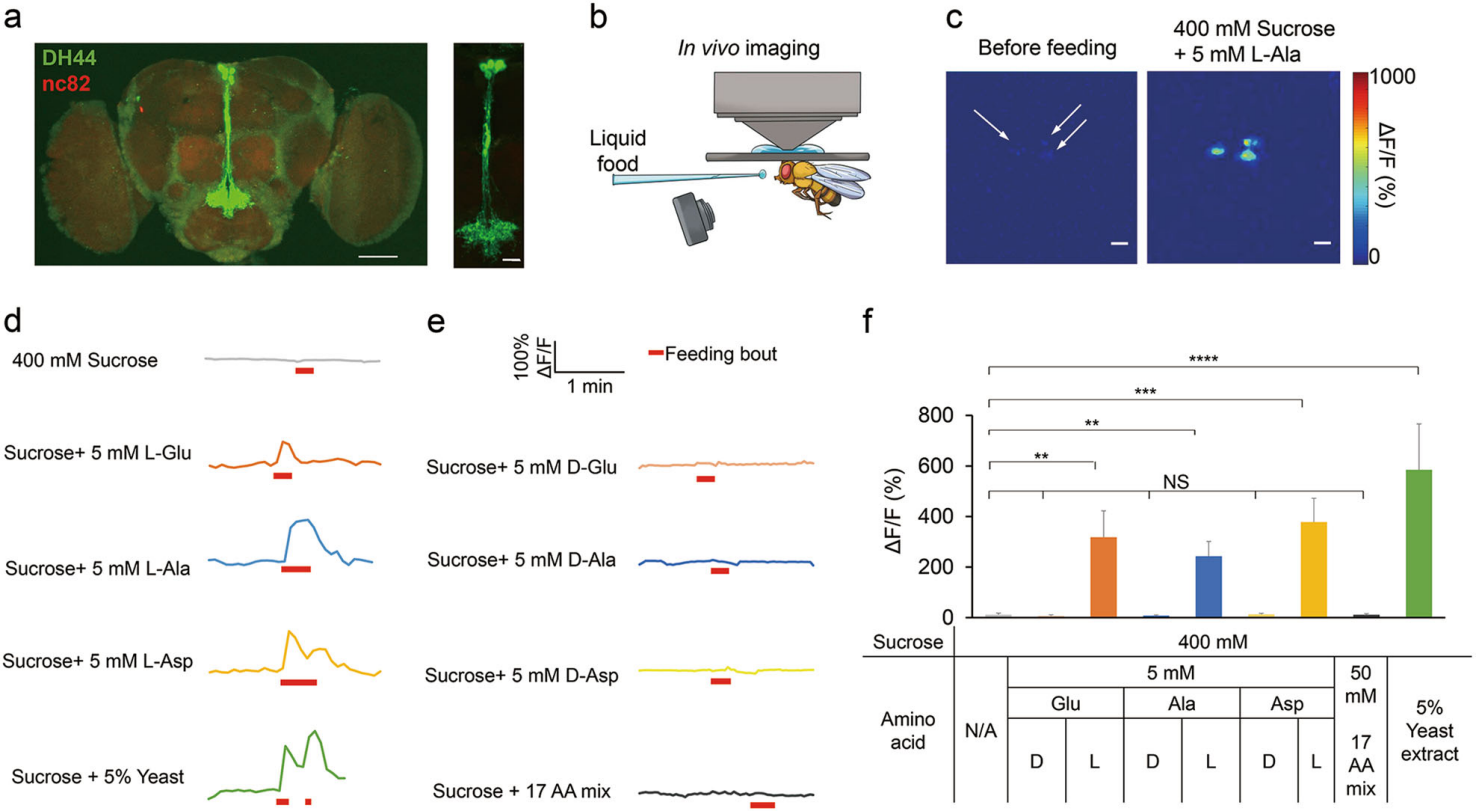
In vivo calcium imaging of DH44^+^ neurons. **a** (Left) The expression of membrane-bound GFP (mCD8GFP) in DH44^+^ neurons driven by *DH44-GAL4*. Scale bar, 20 μm. (Right) An enlarged image of the PI region seen on the left. Scale bar, 10 μm. Green: mCD8GFP in DH44^+^ neurons. Red: nc82. **b** Schematic diagram of in vivo calcium imaging. **c** The in vivo calcium responses of DH44^+^ neurons during a feeding bout with 400 mM sucrose plus 5 mM L-Ala. Scale bar, 10 μm. **d** Representative traces of in vivo calcium responses of DH44^+^ neurons during feeding bouts with 400 mM sucrose, 400 mM sucrose plus 5 mM L-amino acids, and 400 mM sucrose plus 5% yeast extract. **e** Representative traces of in vivo calcium responses of DH44^+^ neurons during feeding bouts with 400 mM sucrose plus 5 mM D-amino acids, and 400 mM sucrose plus 50 mM 17-AA mix. **f** Quantification of the in vivo calcium responses of DH44^+^ neurons to the indicated chemicals (*n* = 8–12). Virgin females were used for all experiments shown in this figure. Data are shown as means ± SEM. NS, *P* > 0.05; *P < 0.05; **P < 0.01; ***P < 0.001; ****P < 0.0001

We next sought to examine the role of DH44^+^ neurons in amino acid sensing. DH44 is expressed in only six neurosecretory cells in the pars intercerebralis (PI) region of the fly brain (Fig. 4a).^29^ It has been shown that DH44^+^ neurons could be directly activated by nutritive sugars including D-glucose and D-fructose, suggesting that DH44^+^ neurons are a post-ingestive nutrient sensor in the fly brain.^29^ We thus asked whether DH44^+^ neurons were also responsive to dietary amino acids.

To this end, we first performed in vivo calcium imaging in live flies during their food intake activities (Fig. 4b, c). While feeding the flies with sucrose alone did not elicit calcium responses in DH44^+^ neurons, L-Glu, L-Ala, L-Asp, and yeast extract all strongly activated DH44^+^ neurons (Fig. 4d, f). In contrast, D-Glu, D-Ala, D-Asp, as well as a mixture of the other 17 L-amino acids did not elicit calcium responses in DH44^+^ neurons (Fig. 4e, f). Notably, during feeding bouts, DH44^+^ neurons were rapidly activated by L-Glu, L-Ala and L-Asp within only a few seconds after the initiation of food consumption (Fig. 4d). These results strongly suggest that DH44^+^ neurons are a fast sensor to evaluate dietary amino acids.

Since DH44^+^ neurons could also be activated by nutritive sugars such as glucose,^29^ we sought to examine the potential crosstalk between the two types of nutrients, glucose vs. amino acids, in modulating food consumption. We found that like amino acids, glucose was sufficient to promote food consumption in a dose-dependent manner when added to sucrose (Supplementary information, Fig. S6a). Meanwhile, in the presence of glucose, the effects of dietary amino acids to promote food consumption and to activate DH44^+^ neurons were both eliminated (Supplementary information, Fig. S6b, c). These data confirm that nutritive sugars and dietary amino acids both modulate food consumption via a common target, DH44^+^ neurons.

### DH44^+^ neurons are a direct sensor of dietary amino acids

**Fig. 5:**
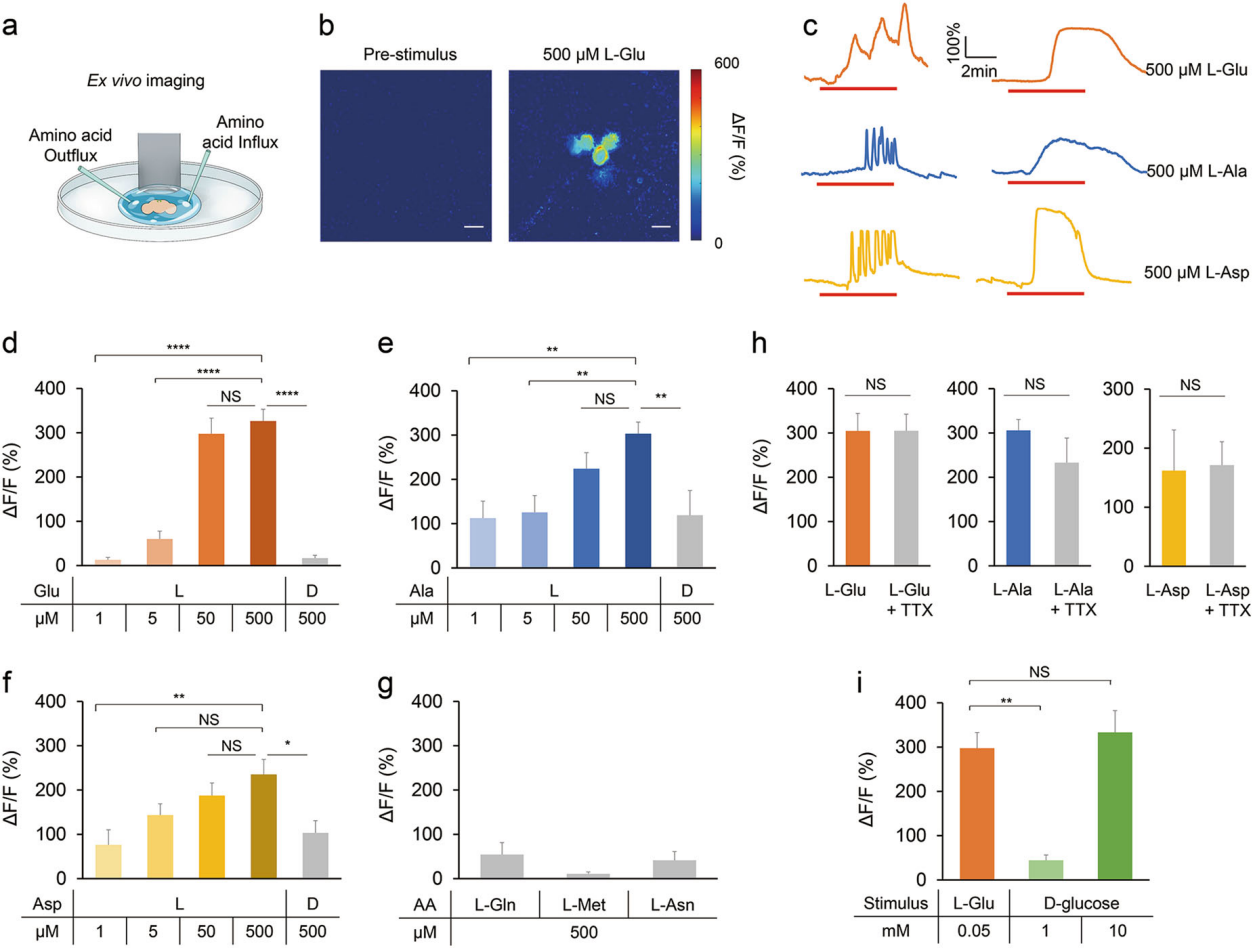
Ex vivo calcium imaging of DH44^+^ neurons. **a** Schematic diagram of ex vivo calcium imaging. **b** The ex vivo calcium responses of DH44^+^ neurons by the perfusion of 500 μM L-Glu. Scale bar, 10 μm. **c** Representative traces of ex vivo calcium responses of DH44^+^ neurons during perfusion of 500 μM L-amino acids. Note that in the ex vivo setup, different DH44^+^ neurons exhibited calcium oscillations or tonic calcium responses upon amino acid stimulation. **d–g** Quantification of the ex vivo calcium responses of DH44^+^ neurons to different concentrations of the indicated amino acids (*n* = 9–40). **h** Quantifications of the ex vivo calcium responses of DH44^+^ neurons to 500 μM of the indicated amino acids in the absence or presence of 1 μM TTX (*n* = 10–14). **i** Quantifications of the ex vivo calcium responses of DH44^+^ neurons to L-Glu and D-glucose (*n* = 10–31). Virgin females were used for all experiments shown in this figure. Data are shown as means ± SEM. NS, *P* > 0.05; *P < 0.05; **P < 0.01; ***P < 0.001; ****P < 0.0001

DH44^+^ neurons could be rapidly activated by dietary amino acids during the feeding bouts. Yet, it was not clear whether such activation solely relied on a novel and fast post-ingestive neural mechanism, or some uncharacterized peripheral amino acid sensors were also involved. To discern between these two possibilities of DH44^+^ neuronal activation, we further performed calcium imaging on isolated fly brains by using an ex vivo preparation, which helped to eliminate any potential gustatory input elicited by amino acids (Fig. 5a, b). Similar preparation has reliably recorded calcium transients in these neurons as described previously.^29,30^ In this ex vivo preparation, we reliably captured two distinct types of calcium transients induced by the influx of amino acids from distinct DH44^+^ neurons, which suggests that there are two intrinsically distinct types of DH44^+^ neurons in each fly brain (Fig. 5c). Consistent with in vivo calcium imaging, perfusion of L-Glu, L-Ala and L-Asp activated DH44^+^ neurons in a dose-dependent manner (Fig. 5d-f). Therefore, amino acids can activate DH44^+^ neurons independent of any potential gustatory input, favoring a rapid post-ingestive pathway for the activation of DH44^+^ neurons.

Furthermore, similar to in vivo imaging results, the D-enantiomers of these three amino acids elicited much lower calcium responses than their L-enantiomers at the same concentration (Fig. 5d-f). It is worth noting that unlike the in vivo results, D-enantiomers still elicited considerable calcium responses in the ex vivo preparations (Fig. 4f vs. Figure 5d-f). One plausible explanation is that the ex vivo preparations are much more sensitive than the in vivo setup, and hence capture the calcium responses from both L-enantiomers and D-enantiomers. Therefore, DH44^+^ neurons are responsive to both L- and D-enantiomers, but with a much reduced sensitivity to D-enantiomers. Also, several L-amino acids that were unable to promote food consumption (Fig. 2a), including L-glutamine (L-Gln), L-methionine (L-Met) and L-asparagine (L-Asn), induced much smaller calcium transients (Fig. 5g).

We suspected that DH44^+^ neurons, like peripheral sensory neurons expressing Ir76b, were directly sensitive to amino acids. In line with this hypothesis, the axonal terminals of DH44^+^ neurons has been found to locate in the gastrointestinal (GI) tract,^29^ making it plausible that these axonal terminals might gain direct access to post-ingestive amino acids. To formally test whether DH44^+^ neurons were directly sensitive to amino acids, we used tetrodotoxin (TTX) to eliminate synaptic inputs of DH44^+^ neurons. As a blocker of voltage-gated Na^+^ channels, TTX blocked synaptic transmission without interfering with the intrinsic calcium transients of neurons.^29,31^ We found that TTX did not affect calcium transients elicited by L-Glu, L-Ala and L-Asp (Fig. 5h). Thus, these results suggest that DH44^+^ neurons can be directly activated by dietary amino acids without synaptic transmissions.

Collectively, DH44^+^ neurons are responsive to two types of nutrients, nutritive sugars^29^ and dietary amino acids. Interestingly, these neurons showed distinct sensitivities to sugars and amino acids in the ex vivo preparation. In DH44^+^ neurons, 0.05 mM L-Glu was sufficient to elicit similar calcium responses to 10 mM D-glucose, which was much higher than calcium transients induced by 1 mM D-glucose (Fig. 5i).

### DH44^+^ neurons mediate the increase in food consumption by dietary amino acids

**Fig. 6:**
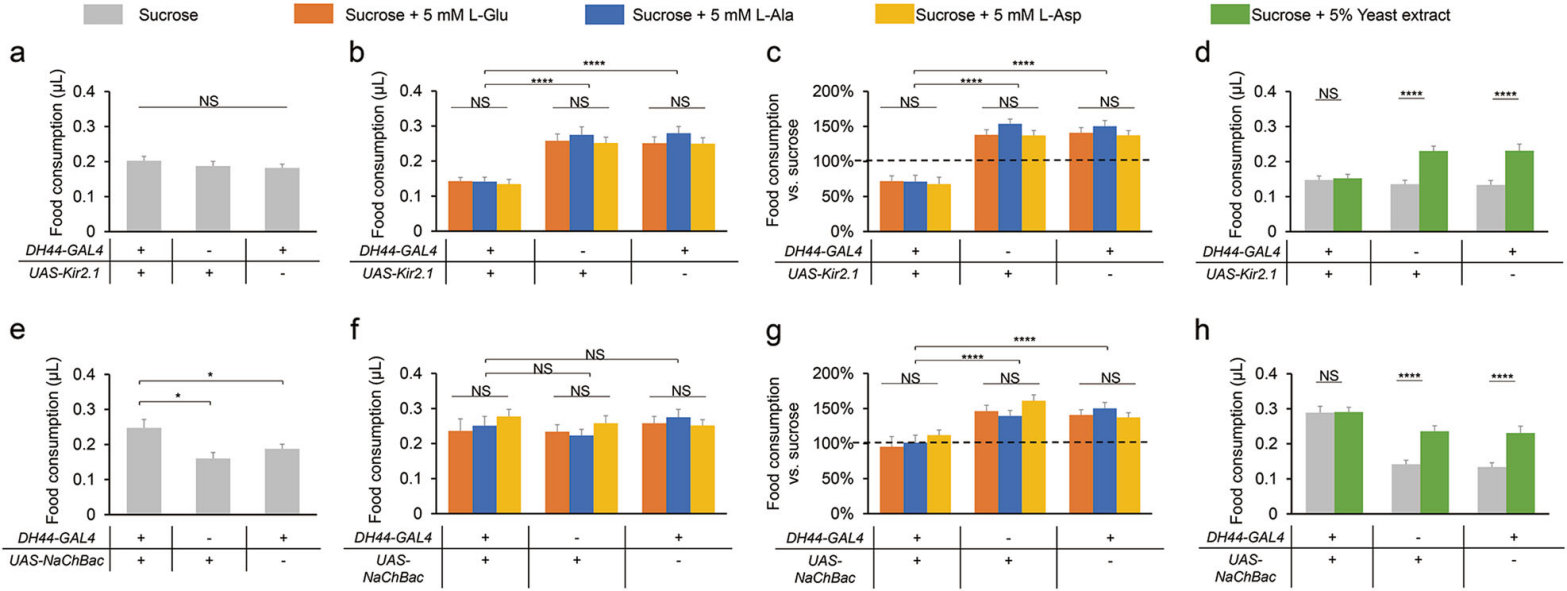
Requirement of DH44^+^ neurons for the increase in food consumption by dietary amino acids and yeast extract. **a, e** Volumes of 400 mM sucrose consumed by flies with the indicated genotypes (*n* = 21–65). **b, f** Volumes of 400 mM sucrose plus 5 mM of the indicated amino acid consumed by flies with the indicated genotypes (*n* = 20–38). **c, g** Changes in food consumption by the addition of 5 mM amino acid compared to 400 mM sucrose alone (dotted line) (*n* = 20–38). **d, h** Volumes of 400 mM sucrose, in the presence or absence of 5% yeast extract, consumed by flies with the indicated genotypes (*n* = 23–36). Virgin females were used for all experiments shown in this figure. Data are shown as means ± SEM. NS, *P* > 0.05; *P < 0.05; **P < 0.01; ***P < 0.001; ****P < 0.0001

The timely sensitivity of DH44^+^ neurons to dietary amino acids suggests that these neurons may be important for controlling amino acid consumption. We sought to test this possibility by directly manipulating the activity of DH44^+^ neurons. Genetic silencing of DH44^+^ neurons by ectopic expression of Kir2.1,^32^ an inwardly rectifying potassium channel, did not affect baseline level of sucrose consumption (Fig. 6a). However, silencing DH44^+^ neurons eliminated the increase in food consumption induced by the presence of L-Glu, L-Ala and L-Asp (Fig. 6b, c). Silencing DH44^+^ neurons also eliminated the effect of yeast extract to promote feeding (Fig. 6d). Taken together, DH44^+^ neurons are required for dietary amino acid intake of fruit flies. Consistent with this conclusion, acute silencing of DH44^+^ neurons by employing a temperature-sensitive *tub-GAL80*^*TS*^ exerted similar effect on amino acid-induced food consumption (Supplementary information, Fig. S7).^33^

It is worth noting that dietary amino acids modestly suppressed amino acid consumption when DH44^+^ neurons were silenced (Fig. 6c). These results indicate that besides DH44^+^ neurons, amino acids may be sensed by additional neural pathway(s) which imposes an inhibitory effect on food consumption. One possible candidate is the insulin signaling pathway that conveys satiety signals.^9^ Evidently, neuronal knockdown of the *Drosophila* insulin-like receptor (dInR) might increase food consumption in the presence of amino acids (Fig. 3a, last column).

To test whether activation of DH44^+^ neurons could enhance food consumption independent of dietary amino acids, we used NaChBac, a bacterial sodium channel, to increase the excitability of these neurons.^34^ Artificial activation of DH44^+^ neurons significantly enhanced the consumption of sucrose (Fig. 6e). In the presence of dietary amino acids or yeast extract, DH44^+^ neuronal activation did not further increase food consumption (Fig. 6f-h). Taken together, these results suggest that DH44^+^ neurons mediate the effect of dietary amino acids and yeast extract to promote food consumption.

A previous study has shown that a evolutionarily conserved kinase, general control nonderepressible 2 (GCN2), is required for the detection of EAAD and hence food rejection in larval flies.^11^ Biochemically, GCN2 signaling is activated upon the deprivation of amino acids via uncharged tRNAs.^35,36^ We also asked whether GCN2 signaling was involved in the activation of DH44^+^ neurons by amino acids. Knocking down GCN2 and its downstream target ATF4 (encoded by *cryptocephal* or *crc*) in DH44^+^ neurons both increased the baseline food consumption with sucrose, phenocopying the effect of neuronal activation (Supplementary information, Fig. S8). Therefore, suppression of GCN2 signaling may mediate the effect of dietary amino acids to promote food consumption. To fully confirm the link between GCN2 signaling and amino acid sensing, more detailed mechanism, including how the activity of GCN2 kinase is modulated and how GCN2-ATF4 signaling modulates neuronal activity, remains to be further elucidated.

### CG13248 is required for the increase in food consumption induced by dietary amino acids

We then sought to investigate how dietary amino acids activated DH44^+^ neurons. One possible mechanism is that these amino acids may enter the hemolymph, circulate to the brain, and activate the cell bodies of DH44^+^ neurons in the brain. However, the fast kinetics of DH44^+^ neuronal activation did not support this possibility (Fig. 4d). Meanwhile, mass spectrometry experiments suggested that none of the 20 free amino acids was rapidly increased in the brain after food consumption (Supplementary information, Fig. S9). Therefore, dietary amino acids may activate DH44^+^ neurons via a distinct, local mechanism.

**Fig. 7:**
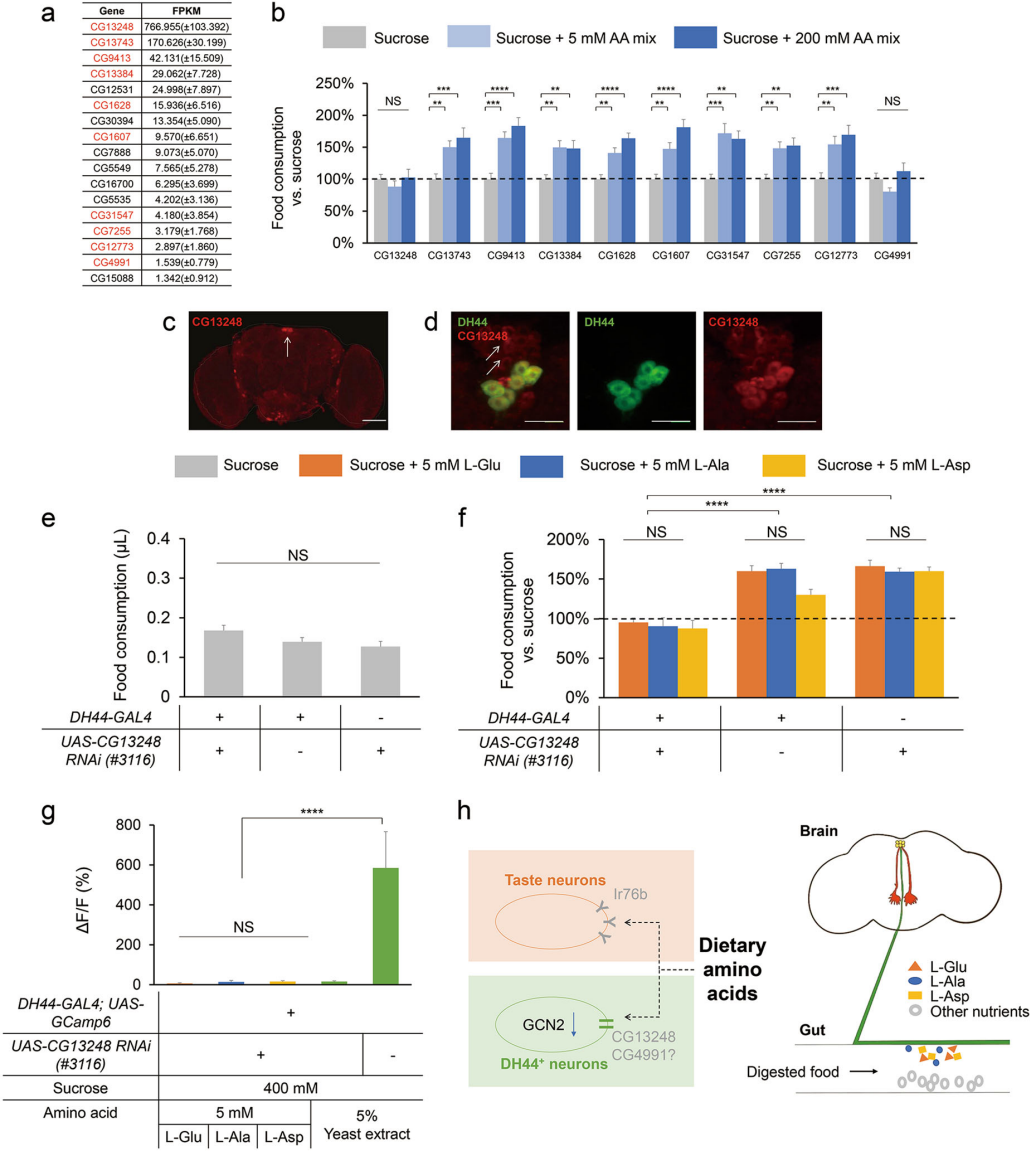
Requirement of CG13248 for the increase in food consumption by dietary amino acids. **a** The list of candidate amino acid transporter genes expressed in DH44^+^ neurons. All genes that are expressed in > 50% of individual DH44^+^ neurons (FPKM ≥ 1) are listed here. Genes in red indicate that we obtained or generated genetic reagents to evaluate their behavioral function. **b** Changes in food consumption by the addition of amino acid mixture compared to 400 mM sucrose alone (dotted line) when the indicated genes are eliminated either by genetic mutation (CG31547) or by RNAi knockdown in DH44^+^ neurons (all other genes) (*n* = 20–23). See Materials and Methods for details. **c** Expression of CG13248 in the fly brain. Scale bar, 20 μm. Red: CG13248. The dotted line indicates the fly brain. The arrow indicates the PI region. **d** Co-localization of CG13248 and DH44 in the PI region of the fly brain. Scale bar, 10 μm. Green: mCD8GFP in DH44^+^ neurons. Red: CG13248. The arrows indicate neurons with CG13248 expression but not DH44 expression. **e** Volumes of 400 mM sucrose consumed by flies with the indicated genotypes (*n* = 21–23). **f** Changes in food consumption by the addition of 5 mM of the indicated amino acid compared to 400 mM sucrose alone (dotted line) (*n* = 20–26). **g** Quantification of in vivo calcium response of DH44^+^ neurons to different food in flies with the indicated genotypes (*n* = 8–10). **h** A working model of dietary amino acid sensing in fruit flies. (Left) Besides a well-documented peripheral sensing mechanism involving Ir76b, the present study proposes a speculative model of a central sensing mechanism, in which DH44^+^ neurons detect L-Glu, L-Ala and L-Asp via a putative amino acid transporter CG13248 and possibly also CG4991. (Right) Anatomically, the cell bodies of DH44^+^ neurons are located in the PI region of the fly brain (yellow), sending their axonal projections to the GI tract (green). Dietary amino acids (colored blocks) may enter DH44^+^ neurites in the gut and activate these neurons. Virgin females were used for all experiments shown in this figure. Data are shown as means ± SEM. NS, *P* > 0.05; *P < 0.05; **P < 0.01; ***P < 0.001; ****P < 0.0001

Given that the axonal terminals of DH44^+^ neurons extend to the fly gut, and that nutritive sugars like glucose may enter DH44^+^ neurons and modulate their activity,^29^ it is possible that nutrients, including nutritive sugars and dietary amino acids, may be in contact with the neurites of DH44^+^ neurons in the GI tract and enter these neurons to modulate behaviors. Therefore, we suspected that for dietary amino acids to activate DH44^+^ neurons, specific amino acid transporter(s) expressed in DH44^+^ neurons might be needed to transport amino acids into these neurons. To identify these amino acid transporter(s), we conducted single-cell RNAseq experiments of individual DH44^+^ neurons following a previously described protocol.^37^ Among ~50 candidate amino acid transporters,^38^ 17 of them showed expression in more than half of the DH44^+^ neurons we examined (Fig. 7a). We obtained genetic mutants or RNAi knockdowns (driven by *DH44-GAL4*) for 10 out of these 17 genes (Fig. 7a, red). Interestingly, we found that eliminating CG13248, the most abundant amino acid transporter expressed in DH44^+^ neurons, blocked the effect of dietary amino acids to promote food consumption (Fig. 7b). Knockdown of CG4991, another amino acid transporter expressed in DH44^+^ neurons, showed similar results in blocking the increase in food consumption induced by dietary amino acids (Fig. 7b).

CG13248 is a putative amino acid transporter.^39^ Antibody against CG13248 revealed its expression in the fly brain, particularly in the PI region (Fig. 7c, arrow). In the PI region, CG13248 was expressed in ~10 neurons per fly brain, including all six DH44^+^ neurons (Fig. 7d). The co-localization of CG13248 and DH44 suggests that CG13248 may be functionally important for DH44^+^ neurons. We knocked down CG13248 expression specifically in DH44^+^ neurons by using two independent RNAi lines (#3116 and #13360.N), which was confirmed by antibody staining (Supplementary information, Fig. S10). Remarkably, knockdown of CG13248 in DH44^+^ neurons completely blocked the effect of L-Glu, L-Ala and L-Asp to promote feeding (Fig. 7f) as well as the calcium responses to dietary amino acids and yeast extract (Fig. 7g). Meanwhile, it had no obvious effect on baseline level of sucrose consumption (Fig. 7e and Supplementary information, Fig. S11). In addition, in line with its potential function for amino acid transport in the GI tract, CG13248 was indeed expressed in neuronal terminals in the gut (Supplementary information, Fig. S12).

In addition, we found that knocking down CG4991 in DH44^+^ neurons had a similar effect to eliminate the calcium responses and the increase in food consumption induced by dietary amino acids (Supplementary information, Fig. S13). Thus, both CG13248 and CG4991 are critical for L-Glu, L-Ala and L-Asp to activate DH44^+^ neurons and to promote food consumption. It is possible that the two putative transporters might act in concert by forming a complex to transport amino acids into cells. The detailed mechanism for the putative transporters needs to be further elucidated.

Notably, L-Glu and L-Asp may also function as neurotransmitters at excitatory synapses and hence activate DH44^+^ neurons. However, we examined the expression of glutamate receptors by single-cell RNAseq and found that no functional glutamate receptor was consistently expressed in DH44^+^ neurons (Supplementary information, Fig. S14). Therefore, it is unlikely that these amino acids activate DH44^+^ neurons in a conventional manner.

## Discussion

In this study, we have used a previously developed feeding assay, named the MAFE assay,^19^ to study the regulation of amino acid consumption in fruit flies. In the MAFE assay, individual flies are immobilized when liquid food is administered directly to their proboscis. As we have previously reported, different components of food intake behavior, including food seeking, feeding initiation and food consumption, are independently regulated.^37,40,41^ Therefore, a unique advantage of the MAFE assay is that it measures food consumption without the interference from the search of food and the initiation of food intake. A potential caveat of the MAFE assay, however, is that flies’ feeding behavior may have a “forced feeding” component since food is delivered directly to their mouthpart. For example, the duration of food consumption seems much shorter in the MAFE assay compared to the CAFÉ assay.^19^ Nevertheless, as we have previously characterized, the “meal” size in the MAFE assay is comparable to that of free-moving flies in the CAFÉ assay.^19,42,43^ Therefore, the findings based on the MAFE assay need to be interpreted with caution and ideally with the confirmation in free-moving flies. Nevertheless, our findings that dietary amino acids promote food consumption, and that DH44 signaling is required for this effect, were both confirmed in free-moving flies.

By using the MAFE assay, we have demonstrated that dietary amino acids significantly promote food consumption, which is further confirmed by the FLIC assay with free-moving flies. It is worth noting that unlike flies’ feeding preference towards yeast and amino acids,^2–5,16,18^ the effect of dietary amino acids to promote feeding is independent of flies’ mating experience and internal nutritional state. The “forced feeding” component may explain why in the MAFE assay, amino acid sensing and the enhanced food consumption are independent of flies’ nutritional state.

Among all 20 natural amino acids, only three of them, L-Glu, L-Ala and L-Asp, but not their D-enantiomers or the other 17 L-amino acids combined, exhibit such an effect in promoting food consumption. Notably, L-Glu, L-Ala and L-Asp are among the most abundant amino acids in yeast, the major protein source of fruit flies.^44^ Therefore, we speculate that the putative sensor of dietary amino acids in flies is tuned to these three amino acids, which provides a reliable indication of protein-rich food sources and facilitates protein intake. Evidently, the detection thresholds of these three amino acids (0.05 mM) are several magnitudes lower than their concentrations in yeast (~30–50 mM)^20^ and in synthetic medium optimized for flies’ lifespan and fecundity (~3–5 mM).^45^

Starting from an RNAi screen, we have established DH44^+^ neurons as a central sensor in the fly brain that detects these three dietary amino acids and promotes food consumption in response. Functional imaging studies reveal that DH44^+^ neurons in the fly brain can be rapidly and directly activated by low concentrations of L-Glu, L-Ala and L-Asp, but not by their D-enantiomers or all other L-amino acids combined. The fast activation of DH44^+^ neurons suits their role to sense dietary amino acids in the time scale of seconds and to increase food consumption in the time course of ~30–40 s. These properties of DH44^+^ neurons may facilitate the search, evaluation, and consumption of desirable protein-rich food sources. Previous reports have identified a small subset of dopaminergic neurons in the adult brain that are involved in the detection of protein deprivation.^1^ Therefore, DH44^+^ neurons and these dopaminergic neurons may act in concert to ensure amino acid homeostasis in fruit flies.

Previous work has shown that DH44^+^ neurons detect nutritive sugars in food including D-glucose and D-fructose.^29^ It is therefore possible that DH44^+^ neurons function as a universal post-ingestive nutrient sensor that evaluates various types of nutrients in the GI tract. Notably, DH44^+^ neurons may employ distinct mechanisms to sense nutritive sugars and amino acids. First, DH44^+^ neurons appear more sensitive to amino acids than to sugars (Fig. 5i). Given that in flies’ food sources, the concentrations of sugars (~50 mM) are also significantly higher than amino acids (~3–5 mM),^45^ this unique property of DH44^+^ neurons may help to ensure the detection of both classes of nutrients in food sources and regulate food intake behavior in response. Second, although both nutritive sugars and amino acids elicit robust calcium responses in DH44^+^ neurons, the sugars induce calcium oscillations,^29^ whereas the amino acids induce two types of responses in different DH44^+^ neurons: calcium oscillations or tonic calcium responses (Fig. 5c). Thus, these results suggest that the sensing mechanisms may differ between sugars and amino acids. It is possible that compared to sugar sensing, additional DH44^+^ neurons are employed for amino acid sensing, which exhibit distinct activation kinetics.

A previous report has shown that nutritive sugars, especially glucose, may enter DH44^+^ neurons via glucose transporter(s) and be metabolized by hexokinase before activating DH44^+^ neurons.^29^ We also sought to understand how specific amino acids activate DH44^+^ neurons. At the molecular level, we have identified CG13248, a putative amino acid transporter that is highly expressed in DH44^+^ neurons. CG13248 is required for the activation of DH44^+^ neurons by amino acids, and for their function in promoting food consumption. It is therefore likely that dietary amino acids may enter these neurons and directly modulate their neuronal activity. These results are consistent with the fact that intracellular GCN2 signaling also plays a role in amino acid sensing. However, it is also possible that CG13248 is not directly involved in amino acid transport as well as neuronal activation. As suggested by a previous report, CG13248 was enriched in neuroendocrine cells in the fly brain and may be involved in the secretory mechanism of neuropeptides.^39^ Based on these results, it is also possible that CG13248 may be involved in the secretion of DH44 from DH44^+^ neurons, downstream of amino acid sensing.

Notably, the axons of DH44^+^ brain neurons project directly to the gut and innervate extensively the GI tract of flies.^29^ It is therefore plausible that dietary nutrients may penetrate the blood brain barrier and enter the axonal terminals of DH44^+^ neurons along the GI tract.^38^ It remains unclear how the axonal terminals of these neurons detect amino acids and how the signals propagate back to the cell bodies. However, it is worth noting that many fly neurons have a pseudo-unipolar property^46^ and hence these gut projections of DH44^+^ neurons might still be responsive to external stimuli, like dorsal root ganglion neurons in the mammalian system.^47^ In addition, neuronal axons can also uptake and retro-transport neurotrophic signals such as BNDF to the cell bodies.^48^ To resolve the possible sensing mechanism, detailed anatomical characterization and finer physiological recordings of DH44^+^ neurons are needed in future studies. It would also be of interest to identify the downstream neurons expressing DH44-R1 and/or DH44-R2 that mediate this behavioral effect.

As strict heterotrophs, rapid detection of amino acids in potential food sources is critical for protein intake. In mammals, amino acid sensing occurs peripherally, by T1R1/T1R3 taste receptor on the oral taste buds.^13,49^ In fruit flies, we propose that two distinct amino acid sensing mechanisms exist (Fig. 7h, left). Peripherally, Ir76b^+^ taste neurons are responsive to several L-amino acids and mediate yeast preference in protein-starved larvae and adults.^15,16,18^ Centrally, we have shown in this study that DH44^+^ neurons in the fly brain detect dietary L-Glu, L-Ala and L-Asp and enhance food consumption. It is also of interest to understand the potential crosstalk between these two mechanisms in regulating protein intake. Notably, Ir76b seems not to be the sole amino acid sensor in fruit flies since protein-starved flies exhibit reduced yet notable yeast preference in the absence of *Ir76b* gene or Ir76b^+^ neurons.^16^ In that case, central sensing of amino acids by DH44^+^ neurons might compensate for loss of peripheral amino acid sensing mediated by Ir76b^+^ neurons to promote protein intake (Fig. 7h).

Mechanically, we propose a speculative model for this central sensing pathway (Fig. 7h, right). Briefly, specific dietary amino acids may reach and enter the axonal terminals of DH44^+^ neurons in the gut via amino acid transporter CG13248 (and CG4991). Upon the entry of these amino acids, DH44^+^ neurons are directly activated via the suppression of GCN2 signaling. The activation of DH44^+^ neurons subsequently enhances the consumption of food sources enriched in amino acids. As we mentioned above, substantial new studies are needed to further examine the details of this working model, e.g., how amino acids reach and enter DH44^+^ neurons, what intracellular signaling pathway mediates the activation of DH44^+^ neurons, and the neural circuitry underlying the modulation of food intake.

The identification of a central amino acid sensor in fruit flies also raises the question of whether similar central amino acid sensing mechanism exists in mammals. Besides the peripheral umami sensing, mammalian brain can detect the presence of free amino acids in the circulation system. For example, specific hypothalamic neurons are responsive to circulating L-Leu via TOR signaling and suppress food intake.^50,51^ In addition, EAAD sensing in rodents is also taste independent and involves the anterior piriform cortex, while the requirement of GCN2 signaling is still under debate.^52–56^ However, unlike DH44^+^ neurons in the fly brain, these known central amino acid sensing mechanisms in mammals play a suppressive role in food intake. It will be of interest to explore the presence of taste-independent mechanism that detects dietary amino acids and promotes food intake in mammals.

## Materials and Methods

### Flies

Flies were kept in vials containing standard fly medium made of yeast, corn and agar at 25 °C and 60% humidity and on a 12-h light-12-h dark cycle. If not otherwise indicated, virgin female flies were collected shortly after eclosion and kept on standard fly medium for 5 days and transferred to medium made of 5% sucrose and 2% yeast extract before being subjected to behavioral experiments. Water-deprived flies were kept in vials with no food or water supply for 18–24 h before behavioral experiments. For experiments involving temperature shift (Supplementary information, Fig. S7), flies were raised at 18 °C for 9 days and transferred to 30 °C for 24 h before the behavioral assays.

All *UAS-RNAi* lines used in the neuropeptide receptor screen (#25858, #25935, #25936, #27275, #27280, #27506, #27509, #27513, #27529, #27539, #28780, #28781, #28783, #29414, #31884, #31958, #35251, #36303, #38346, #38347) and *elav-GAL4* (#25750) were obtained from the Bloomington *Drosophila* Stock Center at Indiana University. For the amino acid transporter screen, the genetic mutant for CG31547 was obtained from the Bloomington *Drosophila* Stock Center at Indiana University (#59219). For other candidate amino acid transporter genes, *UAS-RNAi* lines were obtained from the Tsinghua Fly Center (#3116, #02064.N, #4215, #02284.N, #04347.N, #04422.N, #04964.N, #01189.N2, #13360.N, #13372.N). *DH44-GAL4* was from Yi Rao (Peking University). *UAS-GCN2 RNAi* and *UAS-ATF4 RNAi* were from Pierre Leopold (Université Nice Sophia Antipolis). *Ir76b*^*1/1*^ was from the Bloomington *Drosophila* Stock Center at Indiana University (#51309). *UAS-GCAMP6m* was from the Bloomington *Drosophila* Stock Center at Indiana University (#1399). #13360.N and #13372.N RNAi lines (targeting CG13248 and CG13743, respectively) were newly generated with the RNAi vector system of phiC31 site-specific integration method as previously described with the help from the Tsinghua Fly Center. Genetic mutants were backcrossed for at least 6–8 generations before the behavioral assays. For behavioral experiments using the GAL4/UAS system, the *GAL4* and *UAS* lines were also kept in the same genetic background.

Briefly, a pair of primers (13360.N: CTAGCAGTCAGTGTGATCATCCTGCGATATAGTTATATTCAAGCATATATCGCAGGATGATCACACTGGCG and AATTCGCCAGTGTGATCATCCTGCGATATATGCTTGAATATAACTATATCGCAGGATGATCACACTGACTG; 13372.N: CTAGCAGTATCGGACATTGTGATGGGTTATAGTTATATTCAAGCATATAACCCATCACAATGTCCGATGCG and AATTCGCATCGGACATTGTGATGGGTTATATGCTTGAATATAACTATAACCCATCACAATGTCCGATACTG) were synthesized and generated into hairpin by annealing. The hairpin structure was then imported into V20, a vector that combines the optimized expression features of the previously reported Valium10 for somatic RNAi with a modified scaffold of the microRNA miR-1. Unique cloning sites allow the generation of shRNAs that accommodate the desired sequences, leading to a hairpin with perfect duplex structure, which favors shRNA loading into AGO2, the principal effector of RNAi in flies. Plasmid verified by sequencing was then purified and injected into *Drosophila* embryos. After genetic screening of plasmid information in offspring flies, transgenic germlines were obtained.

*DH44-R1*^*KO*^, *DH44-R2*^*KO*^ and *DH44*^*E1*^ mutant flies were generated by using the CRISPR/Cas9 technique. Briefly, a pair of sgRNA (*DH44-R1*: GTCAATTGTTAGGGGATTCCCCGG and GTTCATAGCATGGAGTTGGTTGG, targeting 14381872-14381894 and 14385243-14385265, respectively; *DH44-R2*: GAAGTGCCAGAGTTCAGGAGTGG and GTTCATTCATAGTGTCCAGTGGG, targeting 12480822-12480844 and 12486608-12486630, respectively; *DH44*: GAATGATGAAAGCCACAGCGTGG and CCACAAGGCTCGTCTGCACGGCC, targeting 9643490-9643512 and 9643544-9643566, respectively) were co-injected together with Cas9 mRNA into *w*^*1118*^ embryos. Homozygous germlines were confirmed by PCR amplification and Sanger sequencing (*DH44-R1*: forward primer-TAAGCCGAGTTCGATGTG, reverse primer-CCATTTGCACATTGAGTTAC; *DH44-R2*: forward primer-CACACTCGTGCCAACTAA, reverse primer-AAGGACGCAGACAGATAAC; *DH44*: forward primer-AAACGGCGGAGGTCAAAGT, reverse primer- CTGTGAGTTCCGTGTTCCATC). Approximately 3.3 kb in *DH44-R1* gene locus (14381876 to 14385244 in Chromosome 2R) and ~5.4 kb in *DH44-R2* gene locus (12480822 to 12486265 in Chromosome 2R) were removed including most of the exons. *DH44*^*E1*^ mutant has a 41-bp deletion (9643542 to 9643582 in Chromosome 3R) in the first exon. All these mutants were viable and showed no apparent morphological and behavioral deficits.

### Chemicals

Sucrose (S7903), agar (A1296), L-alanine (A7627), L-argine (A5131), L-asparagine (A0884), L-aspartate (A8949), L-cysteine (C1276), L-glutamate (G1251), L-glutamine (G3126), L-glycine (G7126), L-histidine (H8000), L-isoleucine (I2752), L-leucine (L8912), L- methionine (M9625), L-phenylalanine (P2126), L-proline (P0380), L-serine (S4500), L-threonine (T8625), L-tryptophan (T0254), L-tyrosine (T3754), L-valine (V0500), L-lysine (L5626), D-alanine (A7377), D-aspartate (V900627), D-glutamate (G1001), denatomium (D5765), and protein A (P6031) were purchased from Sigma-Aldrich. Yeast extract (LP0021) and tryptone (LP0042) were purchased from Thermo Fisher Scientific.

### Behavioral assays

PER was assayed as described previously.^19^ Briefly, individual flies were gently aspirated and immobilized in a 200 μL pipette tip. Flies were first sated with water and then subjected to different tastants with each tastant tested twice. Flies showing PER responses to at least one of the two trials were considered positive to the tastant.

The MAFE assay was performed as described previously.^19^ Individual flies were transferred and mobilized as the PER assay. They were then presented with liquid food filled in a graduated glass capillary (VWR, 53432-604) until they stopped responding to food stimuli for ten serial food stimuli. Food consumption was calculated based on the volume change before vs. after feeding.

The FLIC assay was used to quantify the duration of food contact, as an indirect measure of food consumption, as previously described.^37^ Each food well was filled with liquid food right before the behavioral assays. Flies were transferred into chambers by gentle aspiration and then recorded for 1 h. Electric current > 120 a.u. was counted as a feeding event, as instructed previously.^23^ Durations of consumption were calculated accordingly.

### Calcium imaging

For in vivo imaging, flies carrying *DH44-GAL4*, *UAS-GCaMP6m* and *UAS-RNAi* transgenes if applicable were anesthetized on ice and glued onto transparent tape. Then a hole (~1–2 mm) on the tape was incised to expose the dorsal part of the fly head. The cuticle part around the PI region of the fly brain was gently removed with forceps and the brain was bathed in adult hemolymph-like solution (AHL; 108 mM NaCl, 8.2 mM MgCl_2_, 4 mM NaHCO_3_, 1 mM NaH_2_PO_4_, 2 mM CaCl_2_, 5 mM KCl, 5 mM HEPES, 80 mM sucrose, pH 7.3). A micro manipulator delivered liquid food to the proboscis of the fly at the indicated time and the actual feeding bouts were imaged by a digital camera installed under the imaging stage at 0.5 frame/s (see Supplementary information, Fig. S15). More specifically, at each feeding bout, the flies extended their proboscis to reach the surface of the liquid food and started food ingestion. By adding a blue dye in the liquid food, the actual flow of the dyed food through flies’ pharynx could also be seen. The calcium signals of DH44^+^ neurons were recorded by a Nikon C2 confocal microscope, with a water immersion objective lens (40× /0.80 w DIC N2) at 0.2 frame/s.

For ex vivo imaging, adult fly brains were freshly dissected into the AHL buffer and immobilized with fine pins on a Sylgard-based perfusion chamber. The brain bathed in the AHL buffer was recorded in the first minute to establish a base line. Then the solutions were changed to AHL+ amino acids or other nutrients with the pH adjusted back to 7.3 by gentle perfusion for about 5 min. Solutions in the perfusion chamber were controlled by a valve commander (Scientific Instruments). After stimulation, samples were washed out again with AHL. For the TTX test, 1 μM TTX was added to the AHL solution. All imagings were performed on a LSM 710 Carl Zeiss confocal microscope.

Image analyses were performed in ImageJ and plotted in Excel (Microsoft) or Matlab (MathWorks). The ratio changes were calculated using the following formula: ΔF/F = [F – F_0_]/F_0_, where F is the mean florescence of cell body, F_0_ is the average base line (1-min interval before stimulation).

### Single-cell RNAseq

As described previously,^37^ individual DH44^+^ cells (visualized by GFP expression) were harvested from dissected fly brain under a fluorescence microscope with a glass micropipette pulled from thick-walled borosilicate capillaries (BF120-69-10, Sutter Instruments). Individual cells were immediately transferred to lysate buffer (0.9× PCR Reaction Buffer II, 1.35 mM MgCl_2_, 0.45% NP40, 4.5 mM DTT, 0.18 U/µL SUPERase-In, 0.36 U/µL RNase inhibitor, 12.5 nM UP1 primer, 0.045 mM dNTP mix) and underwent reverse transcription and cDNA amplification (SMARTer Ultra Low RNA Kit for Sequencing, Clonetech).

To build the cDNA library, library preparation kit (NEBNext Ultra II DNA Library Prep Kit, NEB) was used. The amplified cDNAs gained from the former step were sonicated to ~250 bp fragments by the Covaris S2 system, and fragemented cDNAs were then subjected to end-preparation, adaptor ligation, adaptor-ligated DNA cleanup and PCR amplification successively according to the supplier’s protocols. The cDNA libraries were then sequenced by Illumina Hiseq 2500 platform. The sequenced raw data were first pre-processed to remove low-quality reads, adaptor sequences and amplification primer. Reads were mapped to *Drosophila* genome and mapped reads were selected for further analysis. FPKM (Fragments Per Kilobase Of Exon Per Million Fragments Mapped) was used to quantify gene expression.

### Mass spectrometry

Flies starved for 24 h were transferred to 5% sucrose medium in the presence or absence of 5 mM amino acid mixture (see Supplementary information, Fig. S9). Flies were allowed to feed ad libitum for 1 min. Then these flies were executed with liquid nitrogen and their brains were dissected out. The mass spectrometry was performed in the Mass Spec Core of ShanghaiTech University. Briefly, 60 brains were put in a 2 mL conical tube on dry ice and then treated with 1.5 mL of 80% methanol (−80 °C). Brain samples were incubated at −80 °C for 20 min before a 10-min sonication. After incubating for 1 h at −20 °C, the samples were centrifuged at 14,000× *g* for 5 min at 4–8 °C to pellet the tissue debris. The supernatant was transferred to a new tube, added with 500 μL of 80% methanol (−80 °C), and vortexed for 1 min. After another round of 14,000× *g* centrifugation for 5 min at 4–8 °C, the supernatant was transferred to SpeedVac/lyophilize. The dried samples were resolved in 50% methanol and loaded to liquid chromatography analyzer. The results were then analyzed with Analyst TF.

### Immunohistochemistry

Fly samples were dissected in PBS on ice and transferred to 4% PFA for fixation for 55 min. Fixed brains were incubated with Dilution/Blocking Buffer (10% Calf Serum and 0.5% Triton X-100 in PBS) for 1.5–2 h at room temperature and incubated in Dilution Buffer with primary antibodies at 4 °C for 24–36 h. These samples were then washed with Washing Buffer (0.5% Triton X-100 in PBS) for 4× 15 min at room temperature and subsequently incubated with secondary antibodies for 23–24 h at 4 °C. The brains were washed three times with Washing Buffer again before being mounted in Fluoroshield (Sigma-Aldrich). Samples were imaged with Nikon 10× /0.45 and 40× /0.80w. Antibodies were used at the following dilutions: mouse anti-nc82 (1:200, DSHB), rabbit anti-GFP (1:500, Life Technologies), Guinea pig anti-CG13248 (1:1,000, a gift from Paul Taghert), Alexa Fluor 546 goat anti mouse (1:300, Life Technologies), Alexa Fluor 488 goat anti rabbit (1:300, Life Technologies), Alexa Fluor 594 goat anti guinea pig (1:500, Abcam), and Alexa Fluor 488 goat anti guinea pig (1:500, Abcam).

### Statistical analysis

Data presented in this study were all verified for normal distribution by D’Agostino-Pearson omnibus test. Student’s *t*-test (for pairwise comparisons) and one-way ANOVA (for comparisons among three or more groups) were used. If one-way ANOVA detected a significant difference among groups, a post hoc test with Bonferroni correction was performed for multiple comparisons. Two-way ANOVA (and post hoc test if applicable) was applied for comparisons with more than one variant. The scatterplots were shown in Supplementary information, Data S1.

## Electronic supplementary material

Supplementary information, Figure S1

Supplementary information, Figure S2

Supplementary information, Figure S3

Supplementary information, Figure S4

Supplementary information, Figure S5

Supplementary information, Figure S6

Supplementary information, Figure S7

Supplementary information, Figure S8

Supplementary information, Figure S9

Supplementary information, Figure S10

Supplementary information, Figure S11

Supplementary information, Figure S12

Supplementary information, Figure S13

Supplementary information, Figure S14

Supplementary information, Figure S15

Supplementary information, Table S1

Supplementary information, Data S1
