# Supplementary material for: A post-ingestive amino acid sensor promotes food consumption in Drosophila
Source: Cell Res. 2018 Sep 12;28(10):1013–25. doi: 10.1038/s41422-018-0084-9 (PMC6170445; doi:10.1038/s41422-018-0084-9)
Supplement: Supplementary file 1 — Supplementary information, Figure S1 [file 41422_2018_84_MOESM1_ESM.pdf]

Figure S1

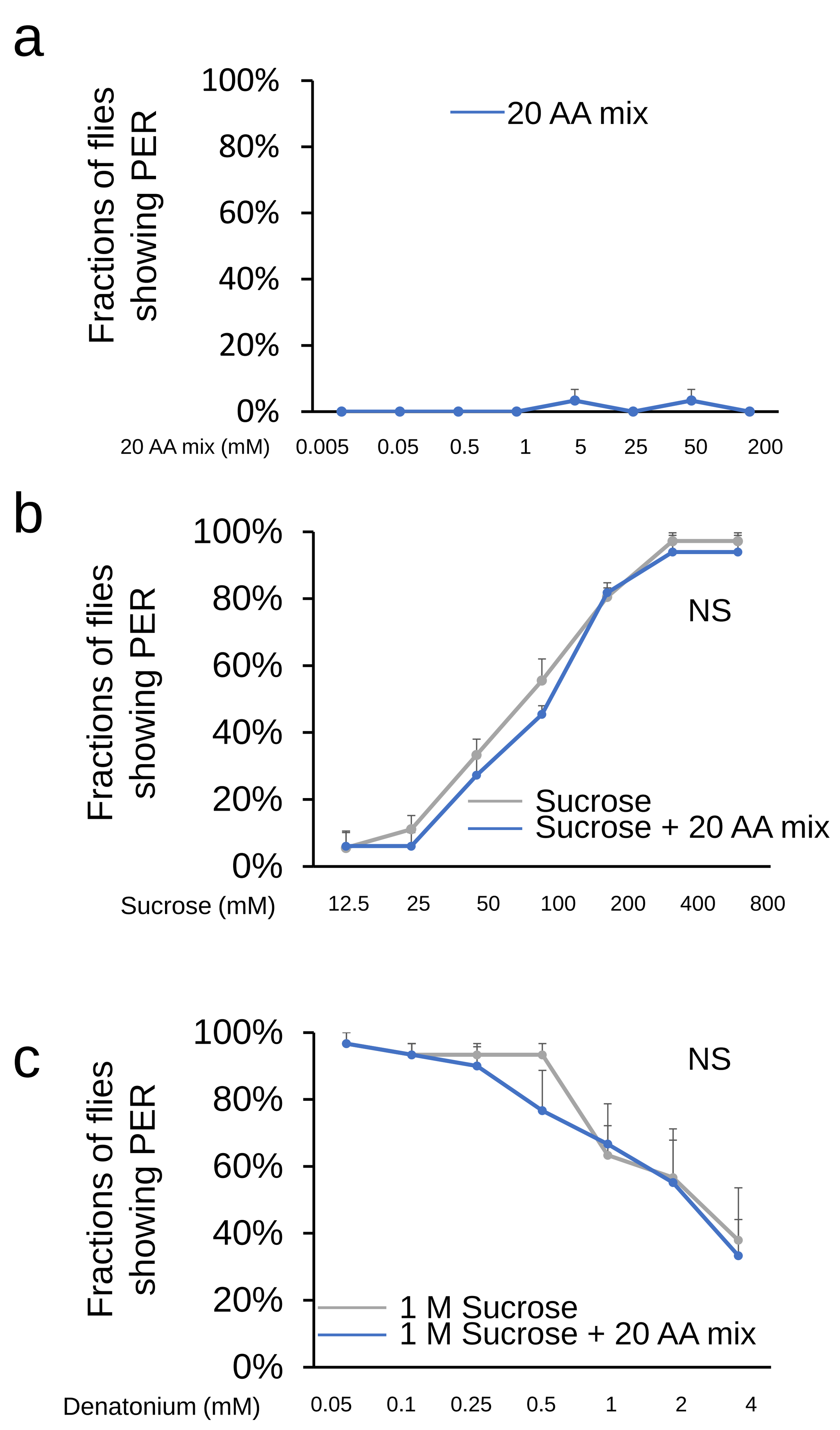
a
100%
80%
60%
40%
20%
0%
Fractions of flies showing PER
20 AA mix
20 AA mix (mM)
0.005
0.05
0.5
1
5
25
50
200
b
100%
80%
60%
40%
20%
0%
Fractions of flies showing PER
NS
Sucrose
Sucrose + 20 AA mix
Sucrose (mM)
12.5
25
50
100
200
400
800
c
100%
80%
60%
40%
20%
0%
Fractions of flies showing PER
NS
1 M Sucrose
1 M Sucrose + 20 AA mix
Denatonium (mM)
0.05
0.1
0.25
0.5
1
2
4

Figure S1. Dietary amino acids do not alter PER responses to sweet and bitter compounds.

(**a**) Fractions of *Canton-S* flies fed *ad libitum* showing PER response to certain concentrations of amino acid mixture (n=21). (**b**) Fractions of *Canton-S* flies fed *ad libitum* showing PER responses to different concentrations of sucrose in the absence or presence of 50 mM amino acid mixture (n=31-38). (**c**) Fractions of *Canton-S* flies fed *ad libitum* showing PER responses to 1 M sucrose plus different concentrations of denatonium in the absence or presence of 50 mM amino acid mixture (n=30-31). Virgin females were used for all experiments shown in this figure. Data are shown as means (± SEM). NS, P > 0.05.
