# Supplementary material for: A post-ingestive amino acid sensor promotes food consumption in Drosophila
Source: Cell Res. 2018 Sep 12;28(10):1013–25. doi: 10.1038/s41422-018-0084-9 (PMC6170445; doi:10.1038/s41422-018-0084-9)
Supplement: Supplementary file 2 — Supplementary information, Figure S2 [file 41422_2018_84_MOESM2_ESM.pdf]

# Figure S2

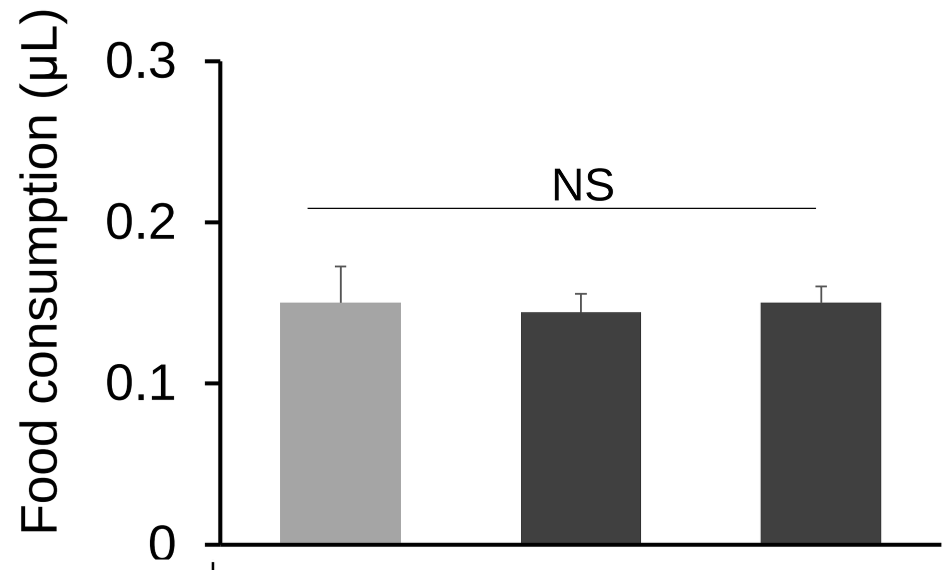


| Sucrose | 400 mM | | |
|---|---|---|---|
| Protein source (1mg/mL) | NA | Protein A | Tryptone |

**Figure S2. Whole proteins do not promote food consumption in the MAFE assay.** Volume of 400 mM sucrose consumed by *Canton-S* flies, in the presence or absence of certain protein sources (n=18-24). Virgin females were used for all experiments shown in this figure. Data are shown as means (± SEM). NS, $P > 0.05$; *$P<0.05$; **$P < 0.01$; ***$P<0.001$; ****$P < 0.0001$.
