# Supplementary material for: A post-ingestive amino acid sensor promotes food consumption in Drosophila
Source: Cell Res. 2018 Sep 12;28(10):1013–25. doi: 10.1038/s41422-018-0084-9 (PMC6170445; doi:10.1038/s41422-018-0084-9)
Supplement: Supplementary file 3 — Supplementary information, Figure S3 [file 41422_2018_84_MOESM3_ESM.pdf]

Figure S3

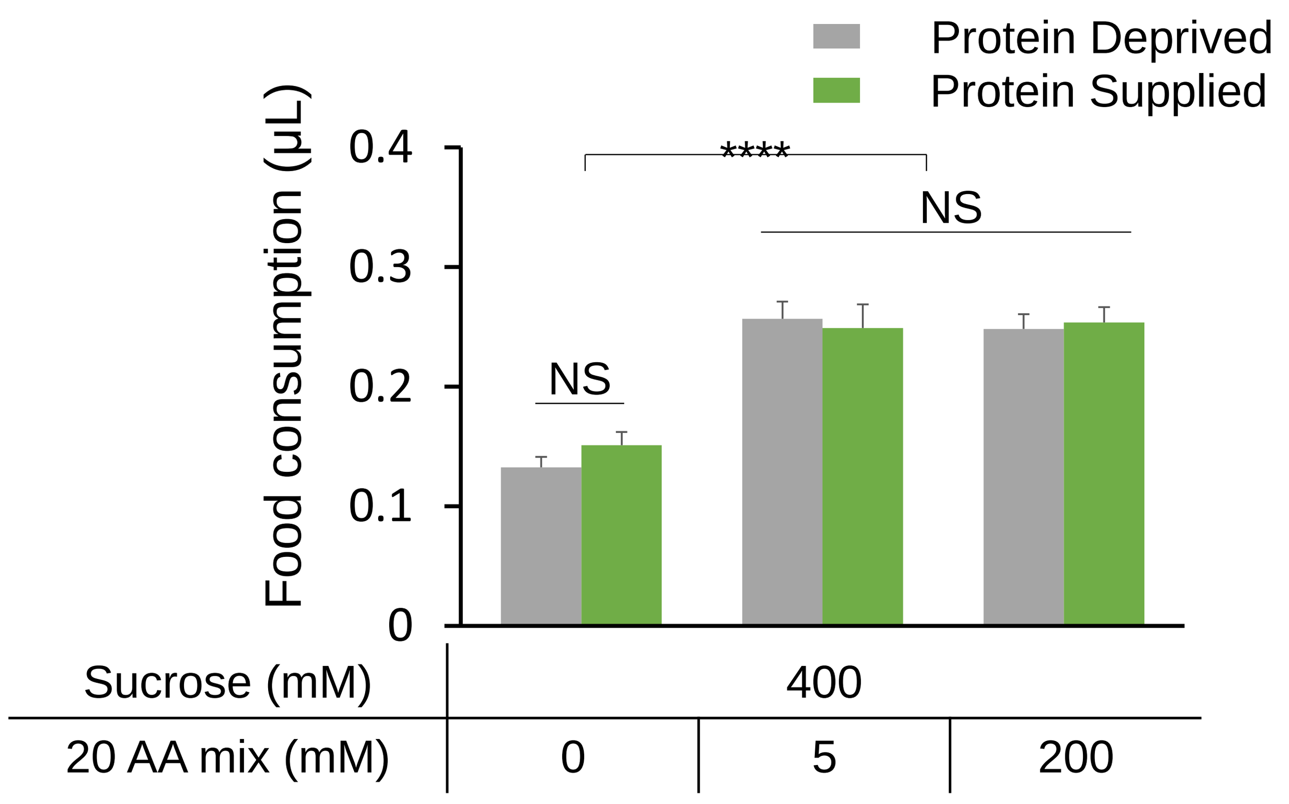
Protein Deprived
Protein Supplied
Food consumption (μL)
0.4
0.3
0.2
0.1
0
****
NS
NS
Sucrose (mM)
400
20 AA mix (mM)
0
5
200

Figure S3. Protein deprivation does not change the effect of dietary amino acids to promote food consumption.

Volume of food consumed by *Canton-S* flies that were raised in the absence of yeast extract (“Protein deprived”) or in the presence of 5% yeast extract (“Protein supplied”) (n=22-59). Virgin females were used for all experiments shown in this figure. Data are shown as means (± SEM). NS, $P > 0.05$; *$P<0.05$; **$P < 0.01$; ***$P<0.001$; ****$P < 0.0001$.
