# Supplementary material for: A post-ingestive amino acid sensor promotes food consumption in Drosophila
Source: Cell Res. 2018 Sep 12;28(10):1013–25. doi: 10.1038/s41422-018-0084-9 (PMC6170445; doi:10.1038/s41422-018-0084-9)
Supplement: Supplementary file 4 — Supplementary information, Figure S4 [file 41422_2018_84_MOESM4_ESM.pdf]

Figure S4

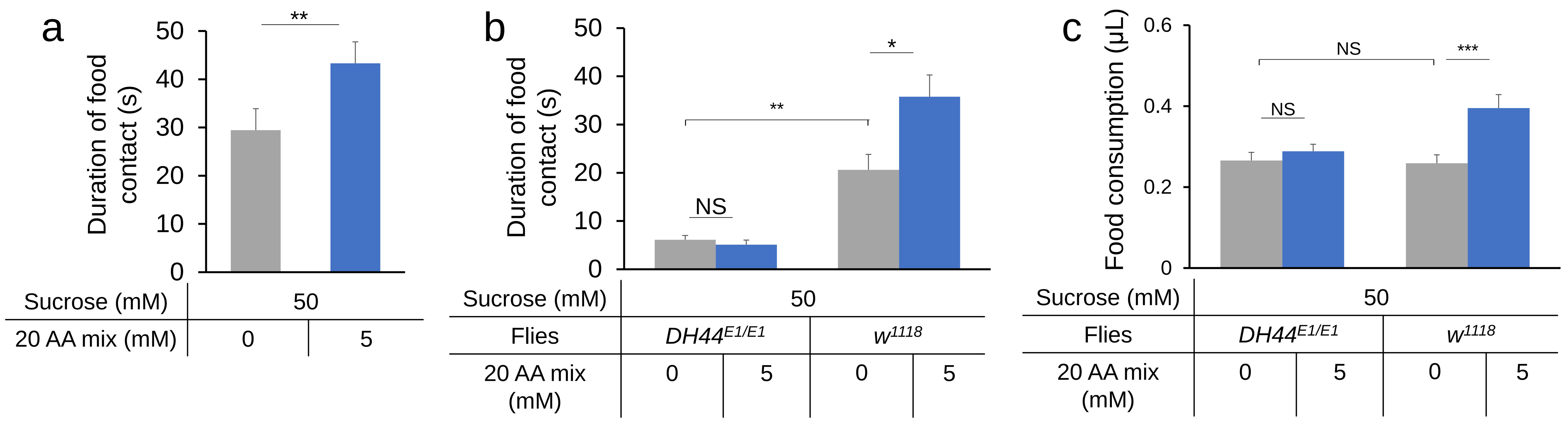
a
Duration of food contact (s)
**
Sucrose (mM)
50
20 AA mix (mM)
0
5
b
Duration of food contact (s)
NS
**
*
Sucrose (mM)
50
Flies
DH44E1/E1
w1118
20 AA mix (mM)
0
5
0
5
c
Food consumption (μL)
NS
NS
***
Sucrose (mM)
50
Flies
DH44E1/E1
w1118
20 AA mix (mM)
0
5
0
5

Figure S4. Dietary amino acids promotes food consumption in free moving flies.

(**a**) Duration of food contact of *Canton-S* flies when fed with sucrose alone (grey) or sucrose plus amino acid mixture (blue) in the FLIC assay (n=29-34). (**b**) Duration of food contact of $DH44^{E1/E1}$ mutant and control flies when fed with 50 mM sucrose in presence (blue) or absence (grey) of 5 mM amino acid mixture (n=19-34). (**c**) Volume of 50 mM sucrose in presence (blue) or absence (grey) of 5 mM amino acid mixture consumed by indicated genotypes (n=21-25). Virgin females were used for all experiments shown in this figure. Data are shown as means (± SEM). NS, P > 0.05; *P<0.05; **P < 0.01; ***P<0.001; ****P < 0.0001.
