# Supplementary material for: A post-ingestive amino acid sensor promotes food consumption in Drosophila
Source: Cell Res. 2018 Sep 12;28(10):1013–25. doi: 10.1038/s41422-018-0084-9 (PMC6170445; doi:10.1038/s41422-018-0084-9)
Supplement: Supplementary file 5 — Supplementary information, Figure S5 [file 41422_2018_84_MOESM5_ESM.pdf]

Figure S5

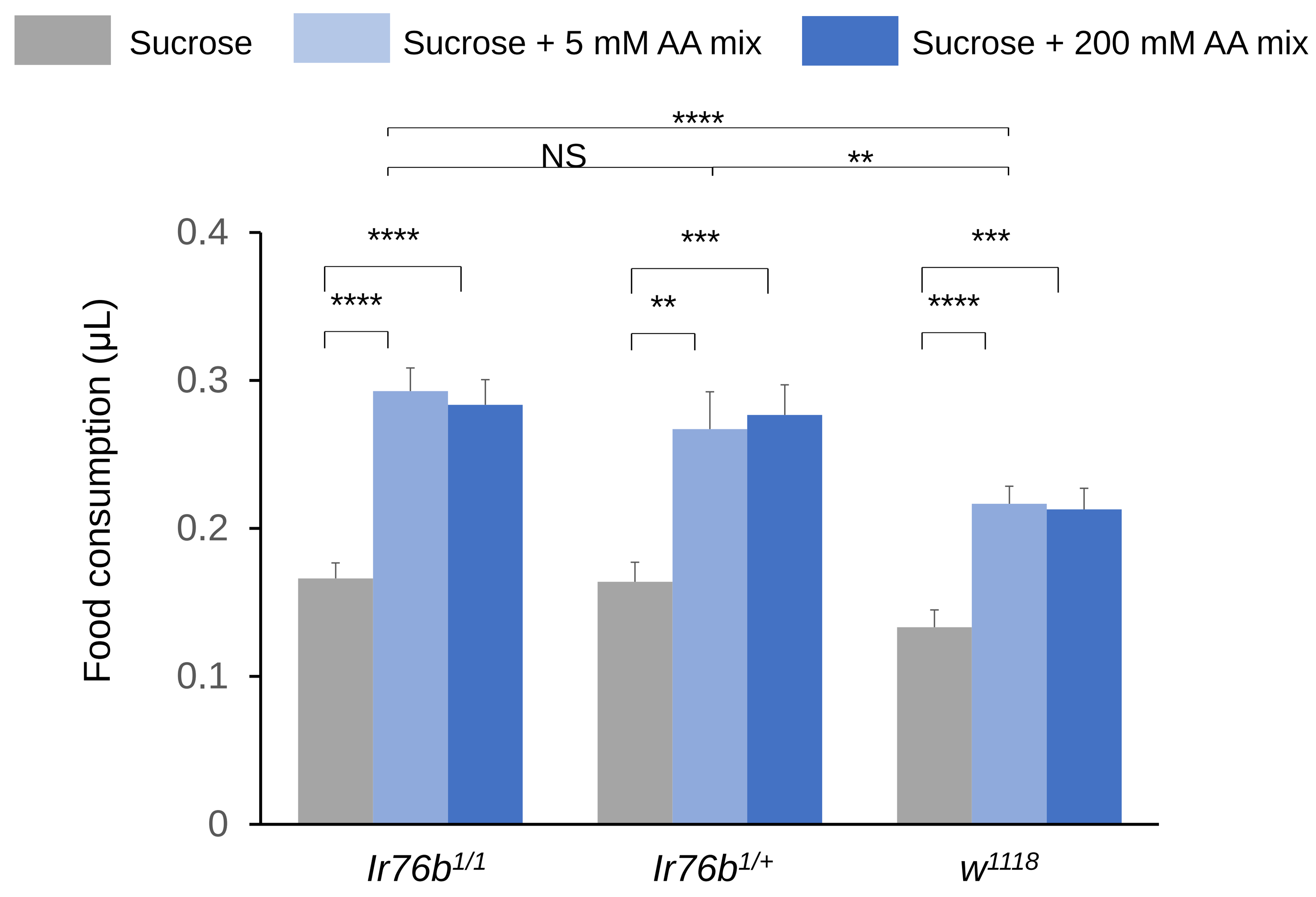

Sucrose
Sucrose + 5 mM AA mix
Sucrose + 200 mM AA mix
****
NS
**
****
***
***
****
**
****
Food consumption (µL)
0.4
0.3
0.2
0.1
0
Ir76b1/1
Ir76b1/+
w1118

Figure S5. Ir76b is not required for dietary amino acids to promote food consumption

Volume of 400 mM sucrose (grey) or 400 mM sucrose plus different concentrations of amino acid mixture (blue) consumed by indicated genotypes (n=20-28). Virgin females were used for all experiments shown in this figure. Data are shown as means (± SEM). NS, $P > 0.05$; *$P<0.05$; **$P < 0.01$; ***$P<0.001$; ****$P < 0.0001$.
