# Supplementary material for: A post-ingestive amino acid sensor promotes food consumption in Drosophila
Source: Cell Res. 2018 Sep 12;28(10):1013–25. doi: 10.1038/s41422-018-0084-9 (PMC6170445; doi:10.1038/s41422-018-0084-9)
Supplement: Supplementary file 6 — Supplementary information, Figure S6 [file 41422_2018_84_MOESM6_ESM.pdf]

Figure S6

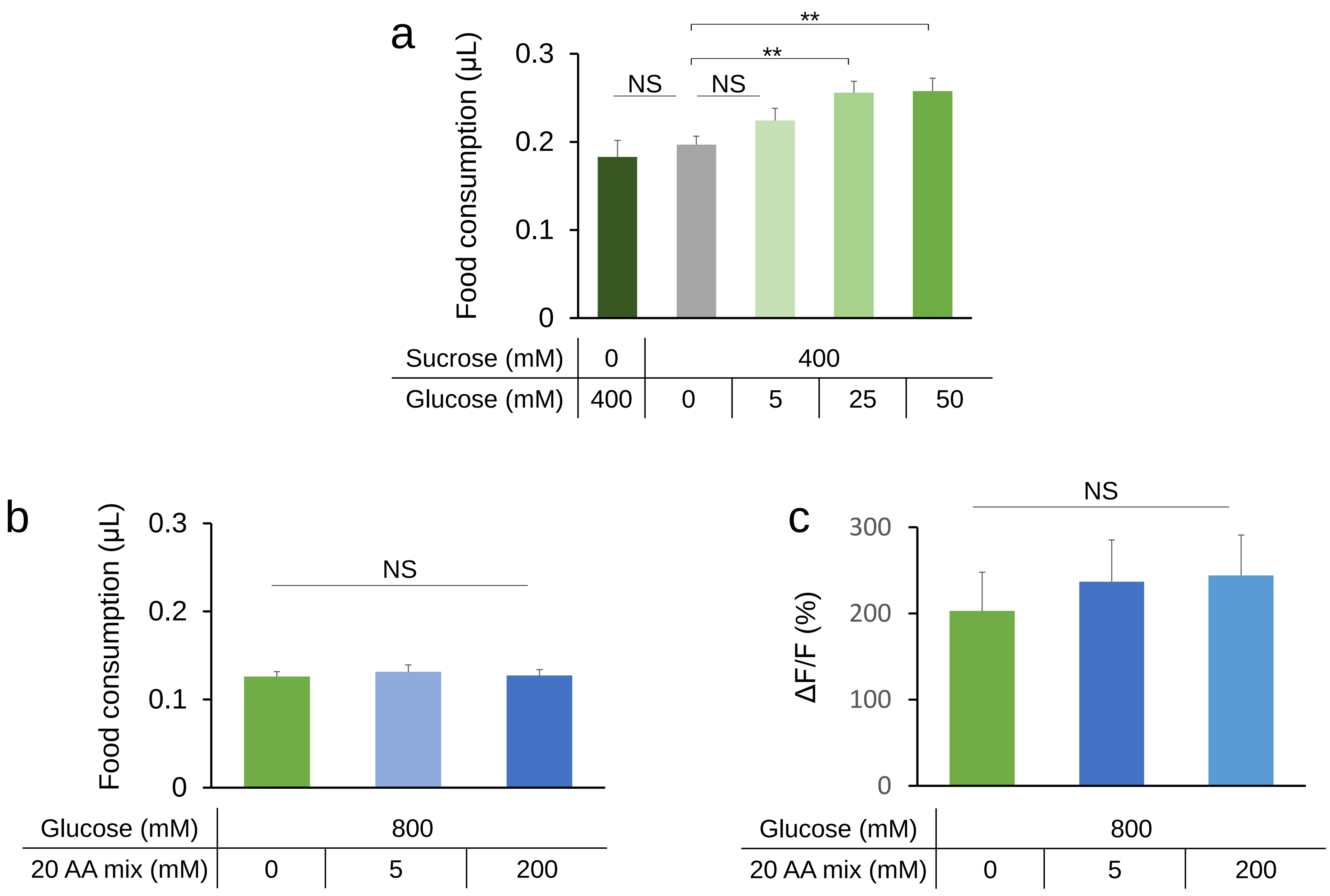
a
Food consumption (μL)
0.3
0.2
0.1
0
**
**
NS
NS
Sucrose (mM)
0
400
Glucose (mM)
400
0
5
25
50
b
Food consumption (μL)
0.3
0.2
0.1
0
NS
Glucose (mM)
800
20 AA mix (mM)
0
5
200
c
ΔF/F (%)
300
200
100
0
NS
Glucose (mM)
800
20 AA mix (mM)
0
5
200

Figure S6. D-glucose and dietary amino acids both promote food consumption.

(a) Volume of 400 mM sucrose consumed by *Canton-S* flies fed *ad libitum* in the presence of varying concentrations of D-glucose (n=25-46). (**b**) Volume of indicated food consumed by *Canton-S* flies fed *ad libitum* (n=22-23). (**c**) Volume of 800 mM D-glucose (green) or 800 mM glucose plus different concentrations of amino acid mixture (blue) consumed by *Canton-S* flies fed *ad libitum* (n=42-45). Virgin females were used for all experiments shown in this figure. Data are shown as means (± SEM). NS, $P > 0.05$; $^{*}P<0.05$; $^{**}P < 0.01$; $^{***}P<0.001$; $^{****}P < 0.0001$.
