# Supplementary material for: A post-ingestive amino acid sensor promotes food consumption in Drosophila
Source: Cell Res. 2018 Sep 12;28(10):1013–25. doi: 10.1038/s41422-018-0084-9 (PMC6170445; doi:10.1038/s41422-018-0084-9)
Supplement: Supplementary file 7 — Supplementary information, Figure S7 [file 41422_2018_84_MOESM7_ESM.pdf]

Figure S7

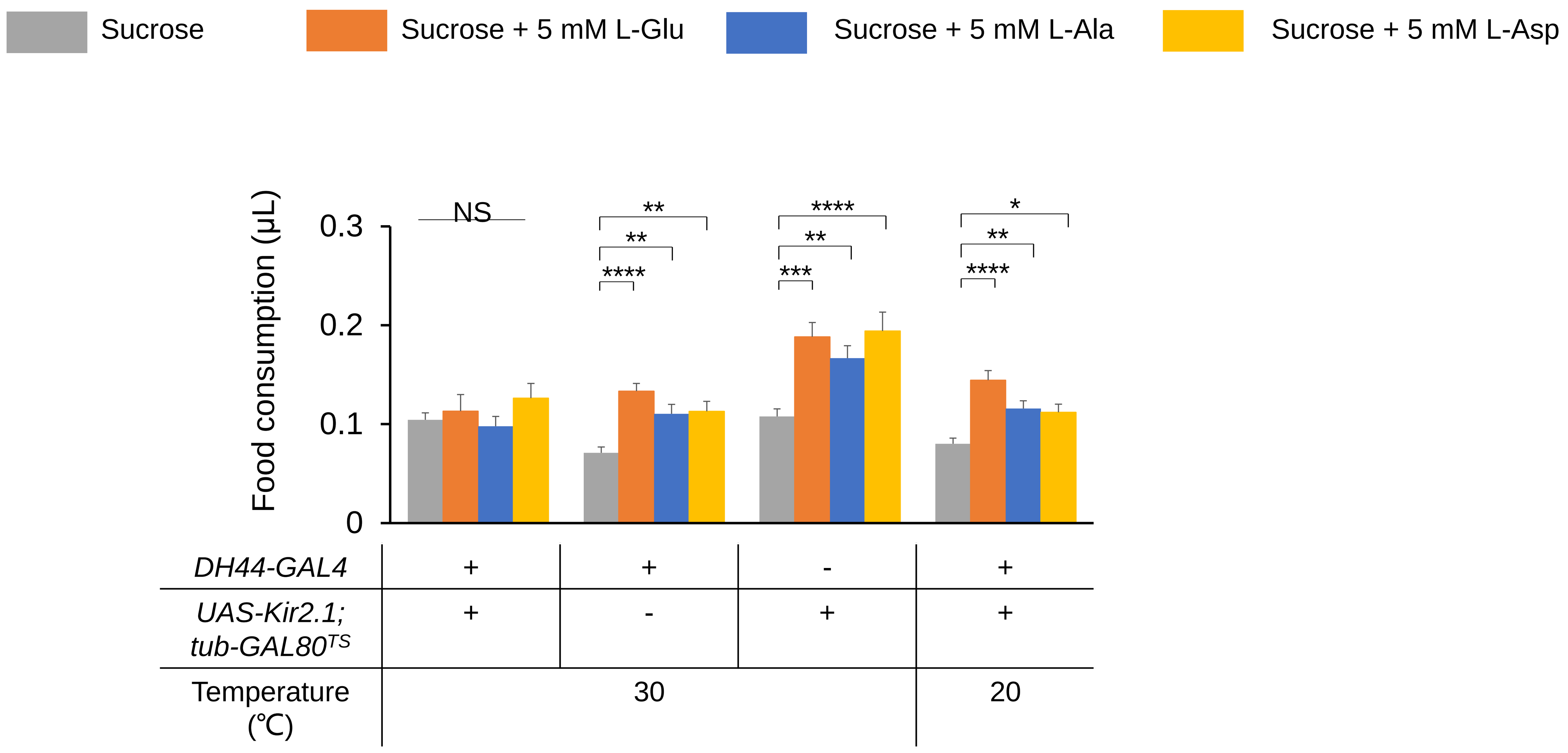
Sucrose
Sucrose + 5 mM L-Glu
Sucrose + 5 mM L-Ala
Sucrose + 5 mM L-Asp
Food consumption (μL)
0.3
0.2
0.1
0
NS
**
**
****
****
**
***
*
**
****
DH44-GAL4
+
+
-
+
UAS-Kir2.1; tub-GAL80TS
+
-
+
+
Temperature (℃)
30
20

Figure S7. Acute silencing of DH44$^+$ neurons eliminates the increase in food consumption by dietary amino acids.

Volume of 400 mM sucrose plus 5 mM of indicated amino acid consumed by indicated genotypes (n=14-31). Virgin females were used for all experiments shown in this figure. Data are shown as means (± SEM). NS, P > 0.05; *P<0.05; **P < 0.01; ***P<0.001; ****P < 0.0001.
