# Supplementary material for: A post-ingestive amino acid sensor promotes food consumption in Drosophila
Source: Cell Res. 2018 Sep 12;28(10):1013–25. doi: 10.1038/s41422-018-0084-9 (PMC6170445; doi:10.1038/s41422-018-0084-9)
Supplement: Supplementary file 8 — Supplementary information, Figure S8 [file 41422_2018_84_MOESM8_ESM.pdf]

Figure S8

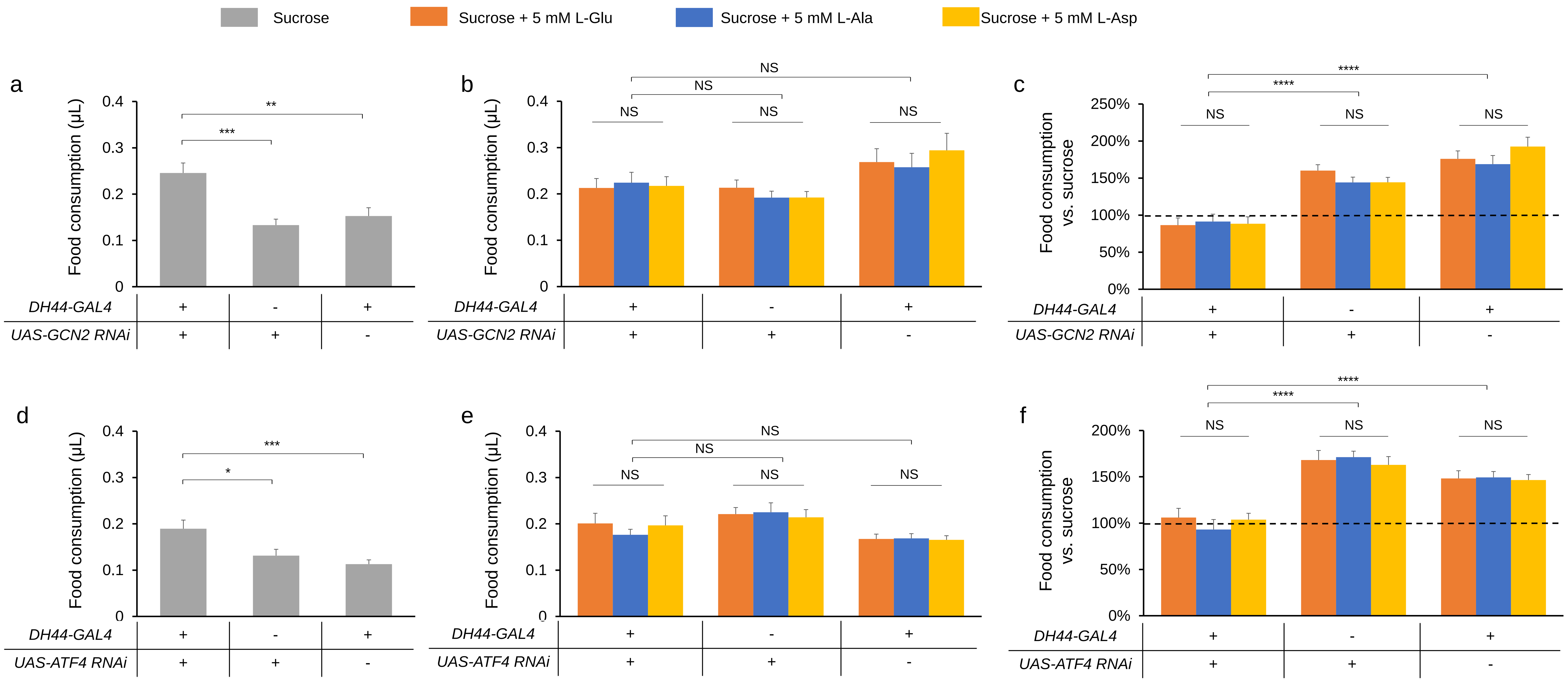

Sucrose
Sucrose + 5 mM L-Glu
Sucrose + 5 mM L-Ala
Sucrose + 5 mM L-Asp
a
b
c
d
e
f
Food consumption (µL)
Food consumption vs. sucrose
NS
**
***
****
*
DH44-GAL4
UAS-GCN2 RNAi
UAS-ATF4 RNAi

Figure S8. GCN2 signaling is required for dietary amino acids to promote food consumption.

(**a, d**) Volume of 400 mM sucrose consumed by indicated genotypes (n=20-24). (**b, e**) Volume of 400 mM sucrose plus 5 mM of indicated amino acid consumed by indicated genotypes (n=19-24). (**c, f**) Changes in food consumption by the addition of 5 mM of indicated amino acid compared to 400 mM sucrose alone (dotted line) (n=19-24). Virgin females were used for all experiments shown in this figure. Data are shown as means (± SEM). NS, P > 0.05; **P<0.01; ***P<0.001; ****P < 0.0001.
