# Supplementary material for: A post-ingestive amino acid sensor promotes food consumption in Drosophila
Source: Cell Res. 2018 Sep 12;28(10):1013–25. doi: 10.1038/s41422-018-0084-9 (PMC6170445; doi:10.1038/s41422-018-0084-9)
Supplement: Supplementary file 9 — Supplementary information, Figure S9 [file 41422_2018_84_MOESM9_ESM.pdf]

Figure S9

AA name	AA-deprived	AA-supplied	P value
ALA	5.05E+04	5.72E+04	0.327
ARG	3.15E+05	3.56E+05	0.339
ASN	4.52E+03	4.82E+03	0.615
ASP	1.98E+03	2.37E+03	0.212
CYS	9.41E+02	8.43E+02	0.389
GLN	7.87E+04	9.43E+04	0.209
GLU	3.24E+04	3.65E+04	0.306
GLY	2.80E+03	3.12E+03	0.404
HIS	7.72E+04	7.60E+04	0.886
LEU	7.33E+03	5.87E+03	0.132
LYS	2.08E+04	2.52E+04	0.14
MET	1.33E+04	1.36E+04	0.824
PHE	4.35E+03	4.42E+03	0.912
PRO	2.13E+05	2.75E+05	0.089
SER	7.13E+03	7.98E+03	0.364
THR	5.05E+03	3.88E+03	0.055
TRP	7.68E+04	6.32E+04	0.152
TRY	2.65E+03	2.86E+03	0.558
VAL	2.79E+05	2.15E+05	0.073

Figure S9. Food consumption does not increase the levels of free amino acids in the fly brain.

Concentrations of 20 amino acids in the fly brain fed with 5% sucrose (AA-deprived) or with 5% sucrose plus 5 mM amino acid mixture (AA-supplied), examined by mass spectrometry (n=3). Virgin females were used for all experiments shown in this figure.
