# Supplementary material for: A post-ingestive amino acid sensor promotes food consumption in Drosophila
Source: Cell Res. 2018 Sep 12;28(10):1013–25. doi: 10.1038/s41422-018-0084-9 (PMC6170445; doi:10.1038/s41422-018-0084-9)
Supplement: Supplementary file 10 — Supplementary information, Figure S10 [file 41422_2018_84_MOESM10_ESM.pdf]

Figure S10

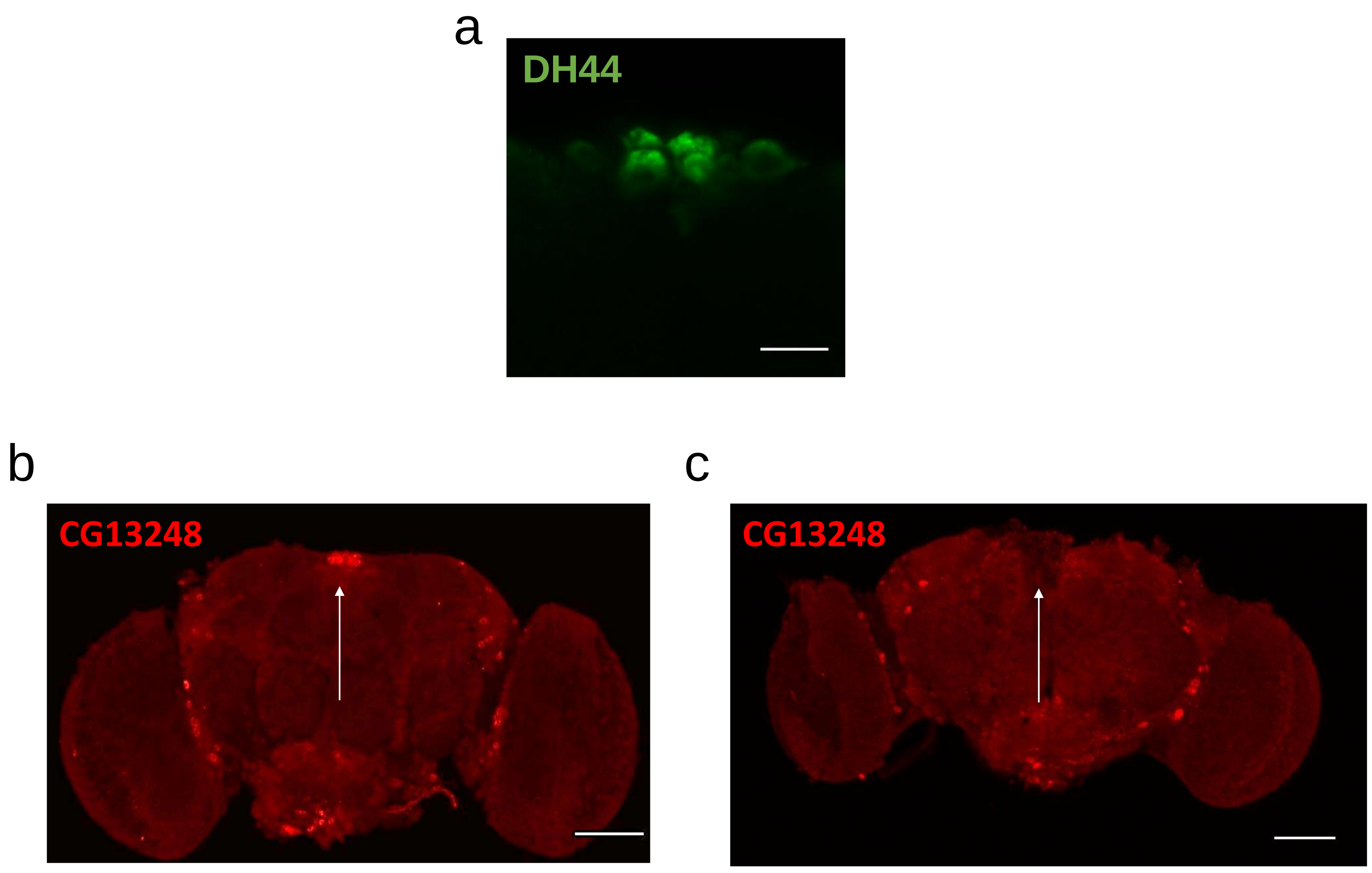

Figure S10. Validation of CG13248 RNAi knock-down in DH44$^+$ neurons.

(a) The expression of GCaMP in DH44$^+$ neurons after CG13248 knock-down, suggesting these cells are still intact and alive. The scale bar represents 10 μm. (**b-c**) The expression of CG13248 visualized by CG13248 antibody, in the brain of control (**b**) and CG13248 knock-down flies (**c**). The scale bar represents 20 μm. Arrows indicate DH44$^+$ neurons in the PI region. Virgin females were used for all experiments shown in this figure.
