# Supplementary material for: A post-ingestive amino acid sensor promotes food consumption in Drosophila
Source: Cell Res. 2018 Sep 12;28(10):1013–25. doi: 10.1038/s41422-018-0084-9 (PMC6170445; doi:10.1038/s41422-018-0084-9)
Supplement: Supplementary file 11 — Supplementary information, Figure S11 [file 41422_2018_84_MOESM11_ESM.pdf]

Figure S11

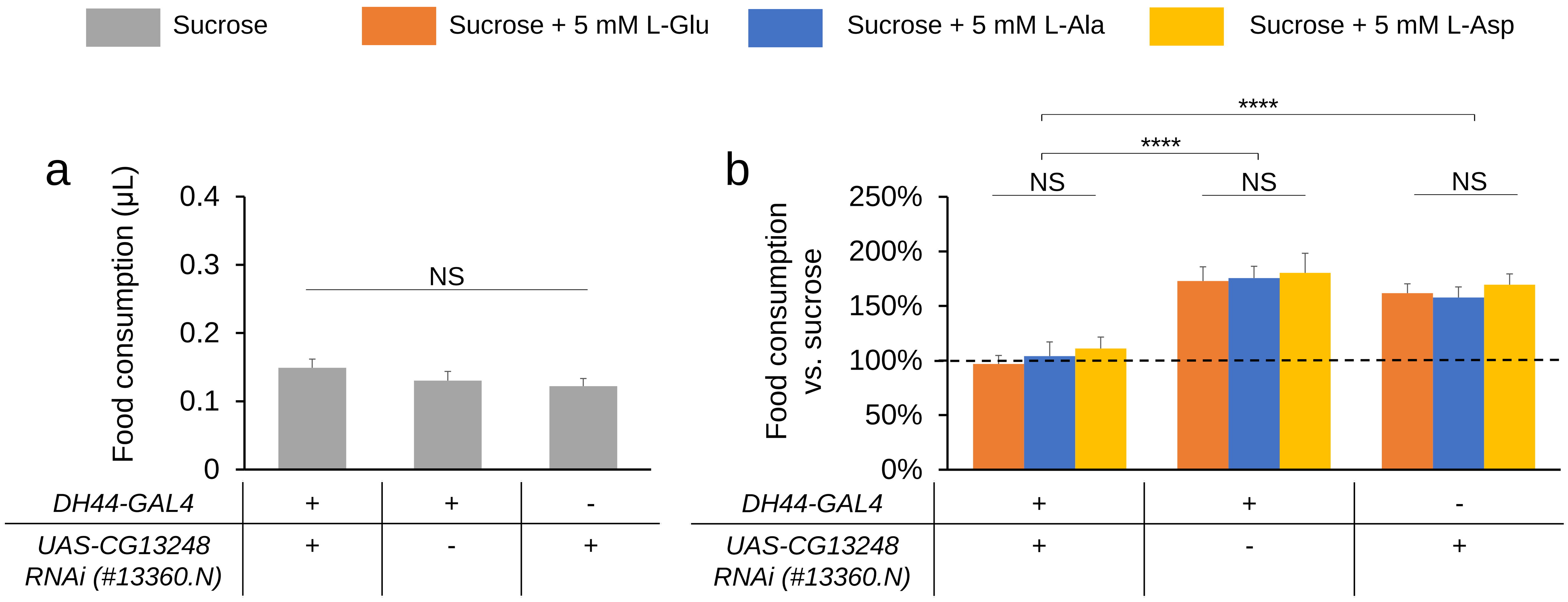

Sucrose
Sucrose + 5 mM L-Glu
Sucrose + 5 mM L-Ala
Sucrose + 5 mM L-Asp
a
Food consumption (μL)
0.4
0.3
0.2
0.1
0
NS
DH44-GAL4
UAS-CG13248 RNAi (#13360.N)
+ + -
+ - +
b
Food consumption vs. sucrose
250%
200%
150%
100%
50%
0%
****
****
NS
NS
NS
DH44-GAL4
UAS-CG13248 RNAi (#13360.N)
+ + -
+ - +

Figure S11. CG13248 is required for the increase in food consumption by dietary amino acids.

(**a**) Volume of 400 mM sucrose consumed by indicated genotypes (n=21-22). (**b**) Changes in food consumption by the addition of 5 mM of indicated amino acid compared to 400 mM sucrose alone (dotted line) (n=21-22). Virgin females were used for all experiments shown in this figure. Data are shown as means (± SEM). NS, $P > 0.05$; *$P<0.05$; **$P < 0.01$; ***$P<0.001$; ****$P < 0.0001$.
