# Supplementary material for: A post-ingestive amino acid sensor promotes food consumption in Drosophila
Source: Cell Res. 2018 Sep 12;28(10):1013–25. doi: 10.1038/s41422-018-0084-9 (PMC6170445; doi:10.1038/s41422-018-0084-9)
Supplement: Supplementary file 12 — Supplementary information, Figure S12 [file 41422_2018_84_MOESM12_ESM.pdf]

Figure S12

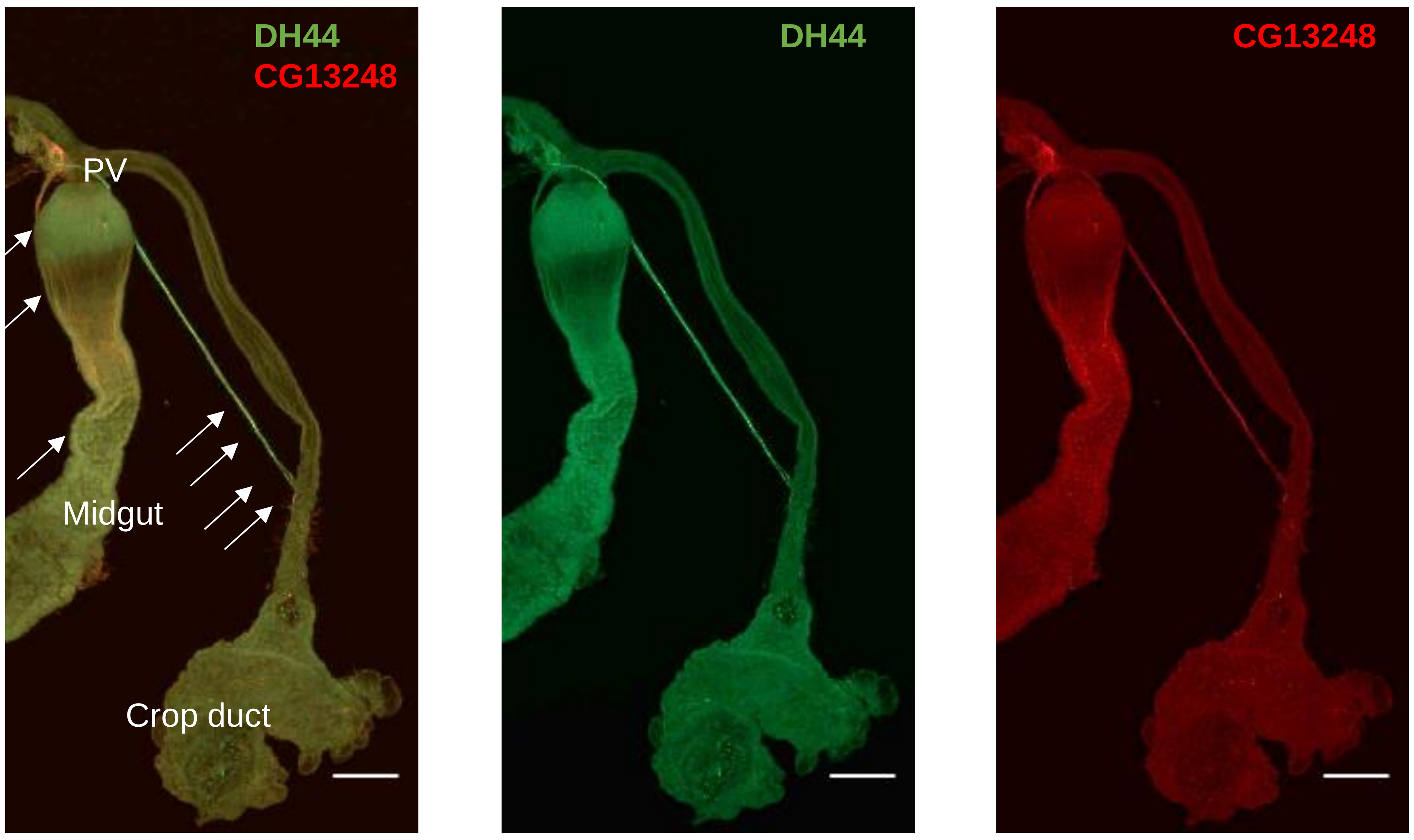

Figure S12. CG13248 is expressed in DH44$^+$ neural terminals in the gut.

As visualized by the antibody, CG13248 (red) is expressed in the DH44$^+$ neural terminals (green) in the gut, innervating both the crop duct and the midgut (arrows). The scale bar represents 50 μm. Virgin females were used for all experiments shown in this figure.
