# Supplementary material for: A post-ingestive amino acid sensor promotes food consumption in Drosophila
Source: Cell Res. 2018 Sep 12;28(10):1013–25. doi: 10.1038/s41422-018-0084-9 (PMC6170445; doi:10.1038/s41422-018-0084-9)
Supplement: Supplementary file 13 — Supplementary information, Figure S13 [file 41422_2018_84_MOESM13_ESM.pdf]

Figure S13

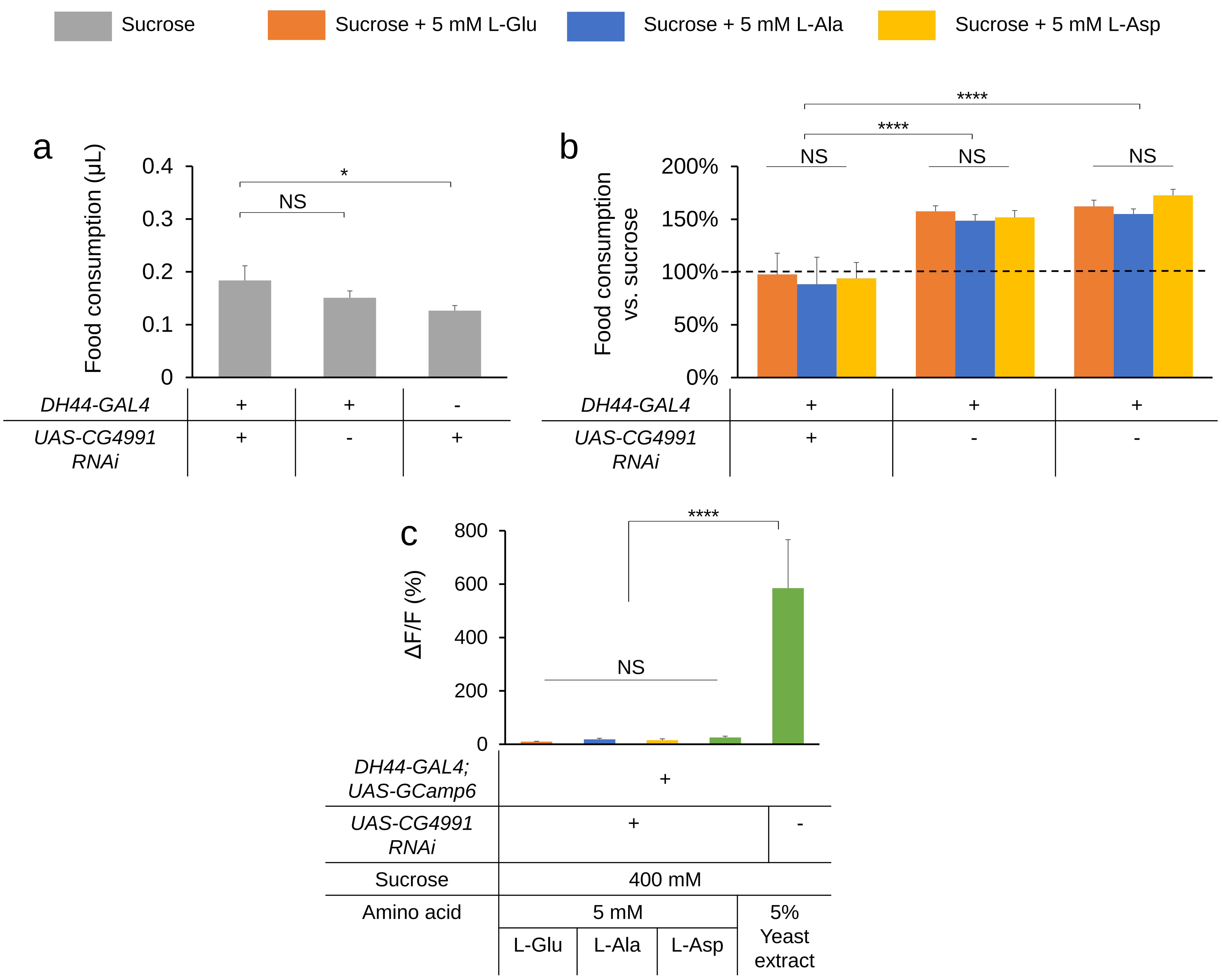
Sucrose
Sucrose + 5 mM L-Glu
Sucrose + 5 mM L-Ala
Sucrose + 5 mM L-Asp
a
Food consumption (μL)
0.4
0.3
0.2
0.1
0
NS
*
DH44-GAL4
+ + -
UAS-CG4991 RNAi
+ - +
b
Food consumption vs. sucrose
200%
150%
100%
50%
0%
NS
NS
NS
****
****
DH44-GAL4
+ + +
UAS-CG4991 RNAi
+ - -
c
ΔF/F (%)
800
600
400
200
0
NS
****
DH44-GAL4; UAS-GCamp6
+
UAS-CG4991 RNAi
+ -
Sucrose
400 mM
Amino acid
5 mM
L-Glu
L-Ala
L-Asp
5% Yeast extract

Figure S13. CG4991 is required for the increase in food consumption by dietary amino acids.

(**a**) Volume of 400 mM sucrose consumed by indicated genotypes (n=19-28). (**b**) Changes in food consumption by the addition of 5 mM of indicated amino acid compared to 400 mM sucrose alone (dotted line) (n=18-30). (**c**) Quantification of *in vivo* calcium response of $DH44^+$ neurons to different food of indicated genotypes (n=8-11). Virgin females were used for all experiments shown in this figure. Data are shown as means (± SEM). NS, $P > 0.05$; *$P<0.05$; **$P<0.01$; ***$P<0.001$; ****$P<0.0001$.
