# Supplementary material for: A post-ingestive amino acid sensor promotes food consumption in Drosophila
Source: Cell Res. 2018 Sep 12;28(10):1013–25. doi: 10.1038/s41422-018-0084-9 (PMC6170445; doi:10.1038/s41422-018-0084-9)
Supplement: Supplementary file 14 — Supplementary information, Figure S14 [file 41422_2018_84_MOESM14_ESM.pdf]

Figure S14

| Glutamate Receptors | Cell #1 | Cell #2 | Cell #3 | Cell #4 | Cell #5 | Cell #6 | Cell #7 | Cell #8 |
|---|---|---|---|---|---|---|---|---|
| GluRIIA | 0 | 0 | 7.8525 | 0 | 0 | 0 | 0 | 0 |
| GluRIIB | 0 | 0 | 0 | 0 | 0 | 0 | 0 | 0 |
| GluRIIC | 0 | 0 | 0 | 0 | 0 | 0 | 0 | 0 |
| Glu-RIA | 0 | 0 | 0 | 0 | 0 | 0 | 0 | 0 |
| Glu-RIB | 0 | 0 | 0 | 0 | 0 | 0 | 0 | 0 |
| NMDAR2 | 0 | 29.6964 | 33.7476 | 2.52333 | 10.9962 | 1.85015 | 14.6972 | 0.602318 |
| NMDAR1 | 0 | 0.727021 | 0 | 25.5672 | 0 | 1.59945 | 12.3314 | 0 |
| mGluRA | 0 | 0 | 0 | 0 | 0 | 0 | 0 | 0 |

**Figure S14. DH44$^+$ neurons do not express functional glutamate receptors.**

FPKM of putative glutamate transporter genes expressed in DH44$^+$ neurons as detected by single-cell RNAseq. Virgin females were used for all experiments shown in this figure.
