# Supplementary material for: A post-ingestive amino acid sensor promotes food consumption in Drosophila
Source: Cell Res. 2018 Sep 12;28(10):1013–25. doi: 10.1038/s41422-018-0084-9 (PMC6170445; doi:10.1038/s41422-018-0084-9)
Supplement: Supplementary file 15 — Supplementary information, Figure S15 [file 41422_2018_84_MOESM15_ESM.pdf]

Figure S15

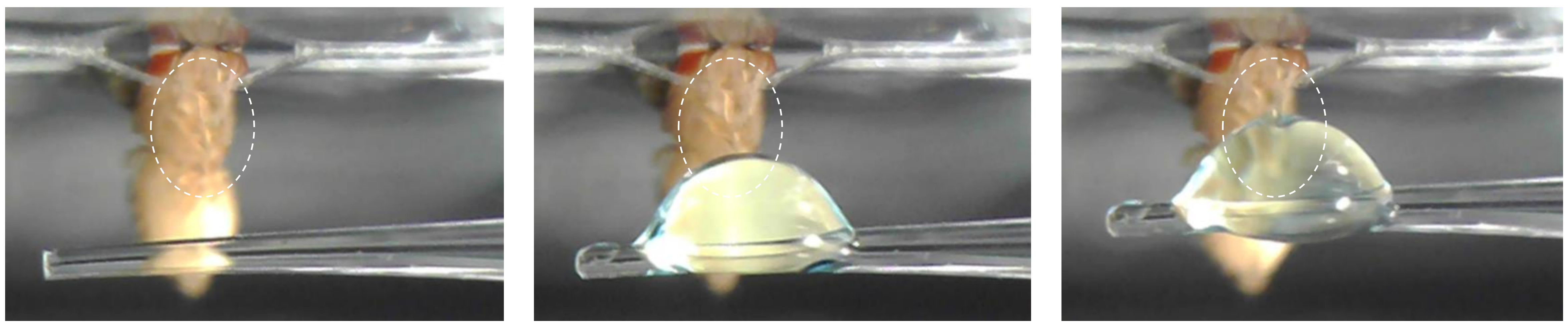

Figure S15. Visualizing food consumption bouts during the *in vivo* calcium imaging. A digital camera was installed underneath the imaging stage to image flies' feeding bouts at 0.5 frame per second. (*left*) Before the liquid food was delivered, the fly's proboscis was retrieved. (*middle*) Liquid food was delivered by the capillary. (*right*) The fly extended its proboscis (in the dashed circle) and started food consumption. The actual flow of the food through flies' pharynx could also been visualized by the addition of a blue dye in the food. Virgin females were used for all experiments shown in this figure.
