# Supplementary material for: A post-ingestive amino acid sensor promotes food consumption in Drosophila
Source: Cell Res. 2018 Sep 12;28(10):1013–25. doi: 10.1038/s41422-018-0084-9 (PMC6170445; doi:10.1038/s41422-018-0084-9)
Supplement: Supplementary file 16 — Supplementary information, Table S1 [file 41422_2018_84_MOESM16_ESM.pdf]

Table S1. Composition of amino acid mixture (5 mM)

L-amino acid	Biological available nitrogen (mM)	Biological available nitrogen (%)
arginine	0.391	7.82
histidine	0.258	5.15
isoleucine	0.248	4.96
leucine	0.353	7.06
lysine	0.267	5.34
methionine	0.007	1.34
phenylalanine	0.153	3.05
threonine	0.296	5.92
tryptophan	0.038	0.76
valine	0.324	6.54
alanine	0.429	8.59
asparagine	0.391	7.82
aspartate	0.162	3.24
cysteine	0.006	0.11
glutamate	0.277	5.53
glutamine	0.553	11.07
glycine	0.305	6.11
proline	0.162	3.24
serine	0.267	5.34
tyrosine	0.067	1.34

Note the concentrations of amino acids in the mixture usually equals to nitrogen concentrations except that for arginine, histidine, asparagine, and glutamine, their concentrations in the mixture are 50% of nitrogen concentrations.

Table S1. Composition of amino acid mixture

Concentrations of each of the 20 L-amino acids in 5 mM amino acid mixture.
