# Supplementary figures and images for: A post-ingestive amino acid sensor promotes food consumption in Drosophila
Source: Cell Res. 2018 Sep 12;28(10):1013–25. doi: 10.1038/s41422-018-0084-9 (PMC6170445; doi:10.1038/s41422-018-0084-9)

**File S1. Scattered plots.**

The scattered plots for all figures in the manuscript.

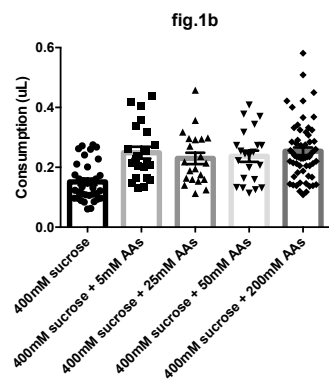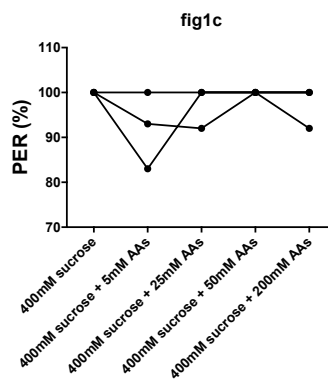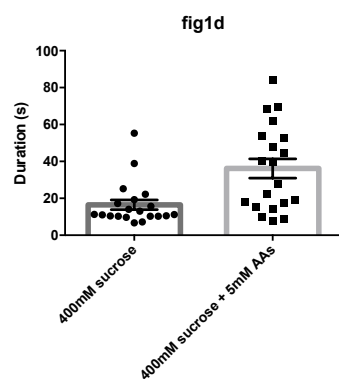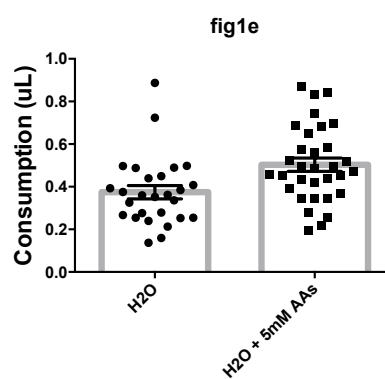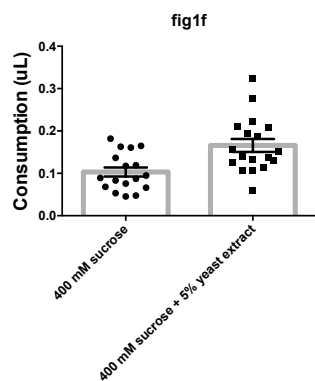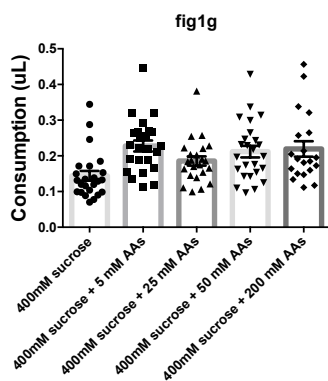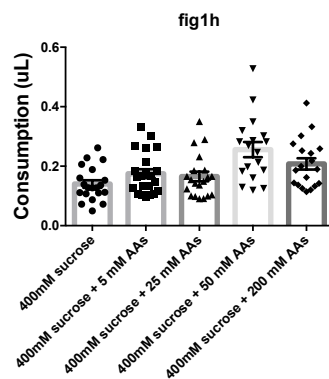

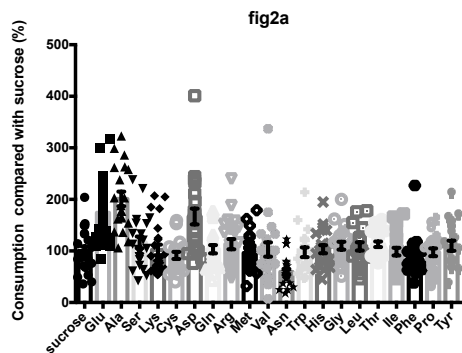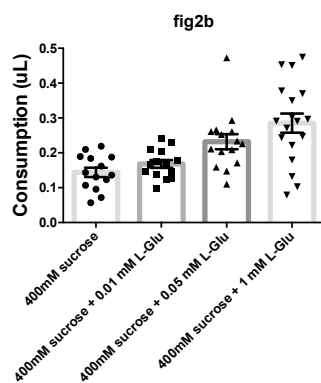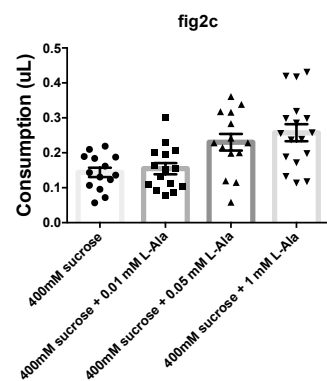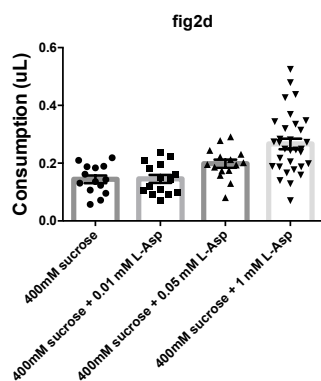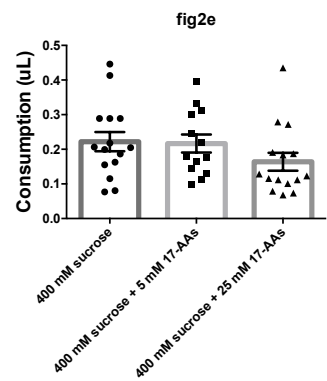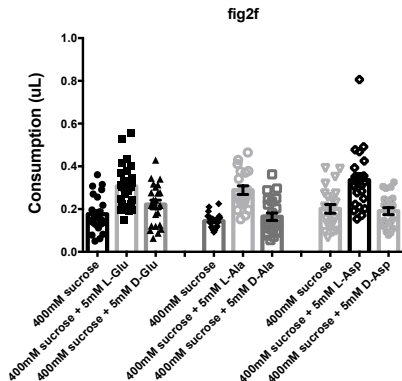

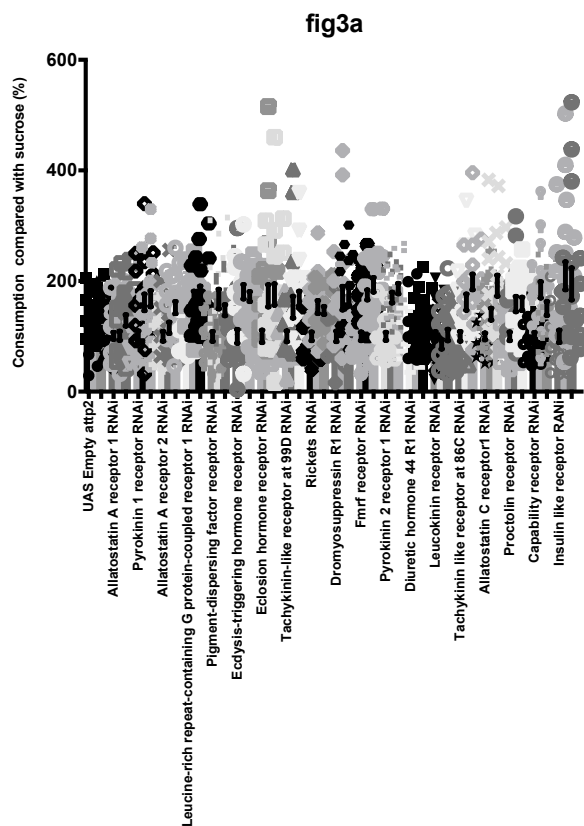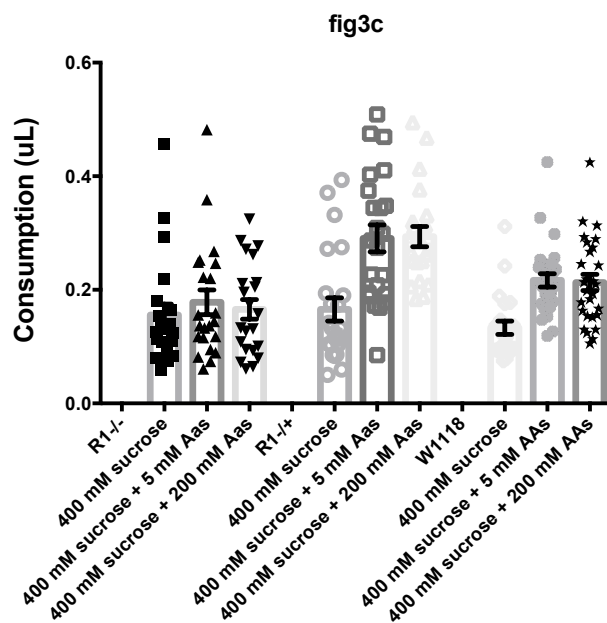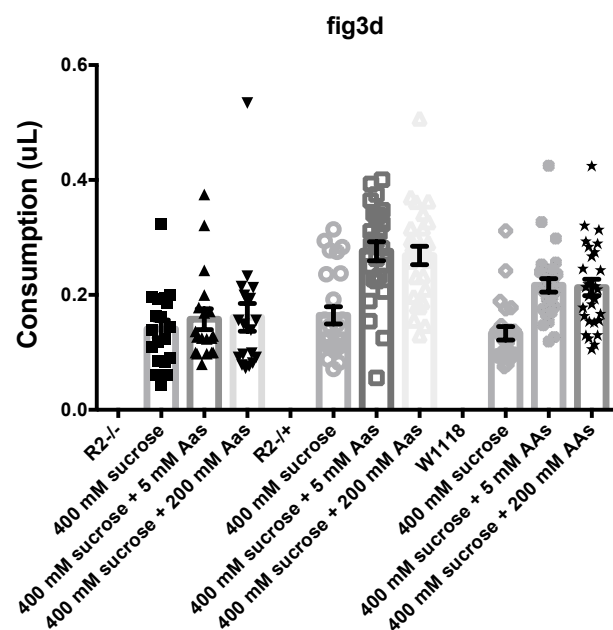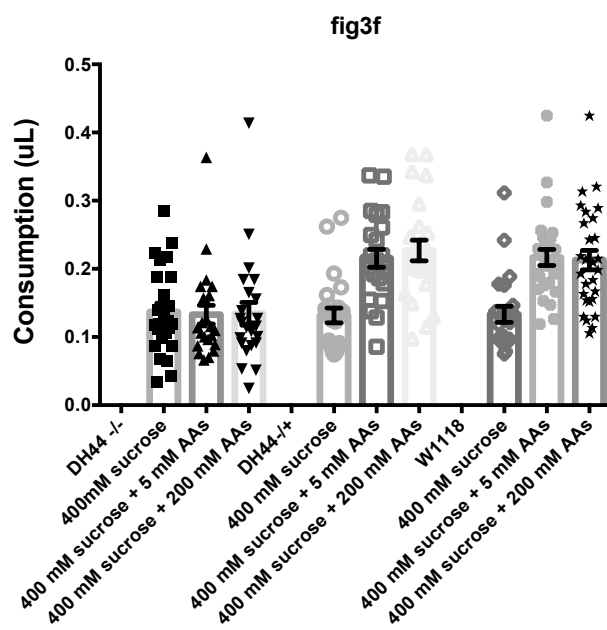

fig4f

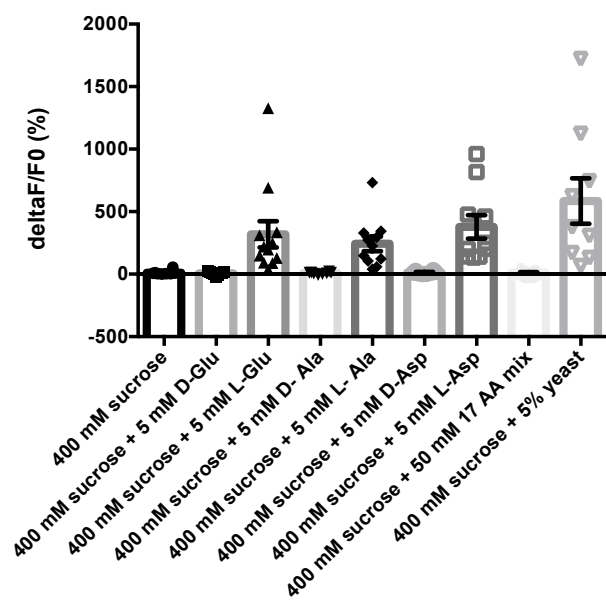

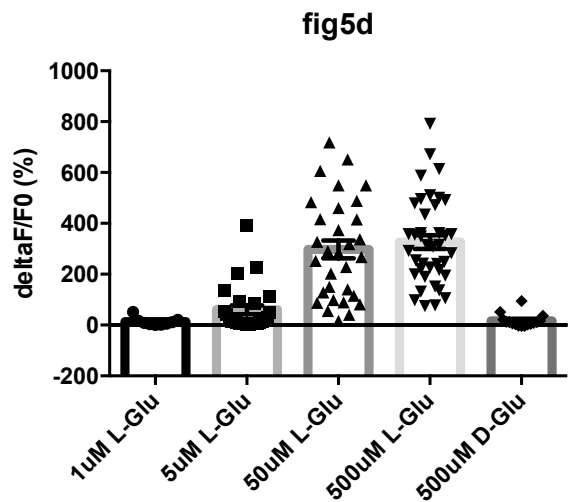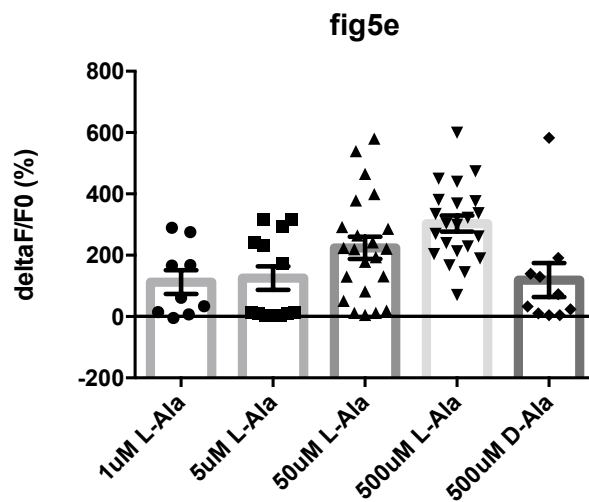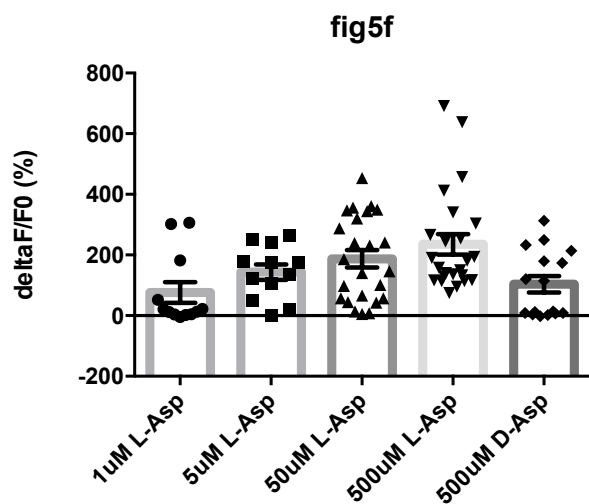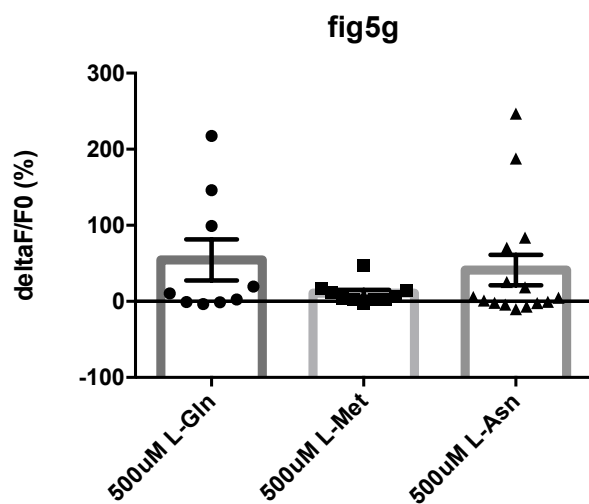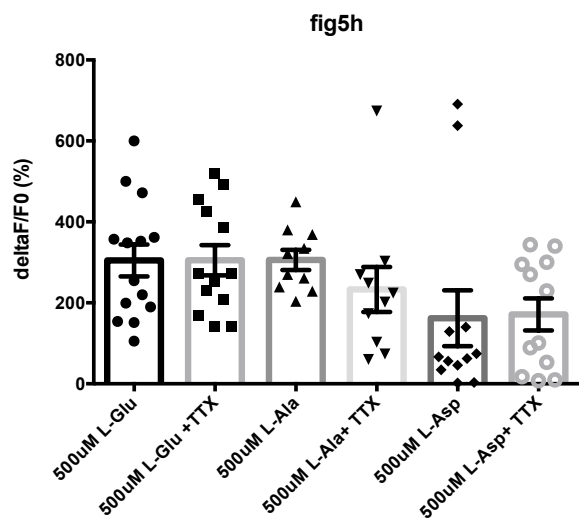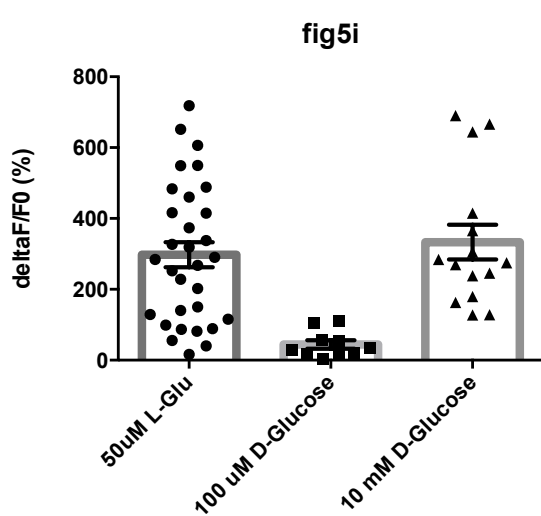

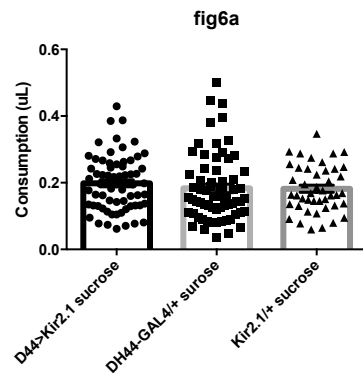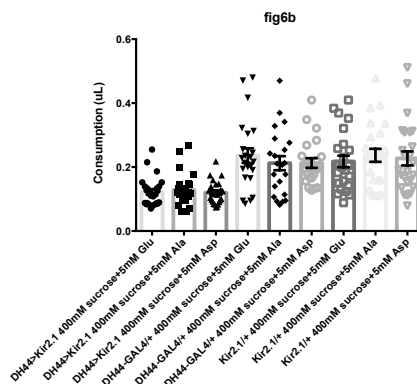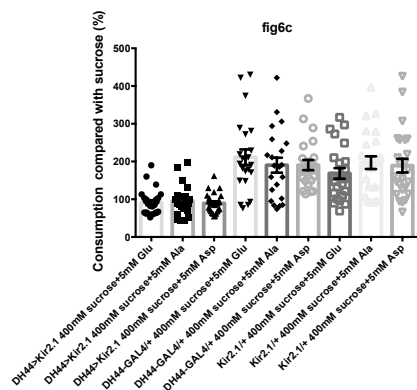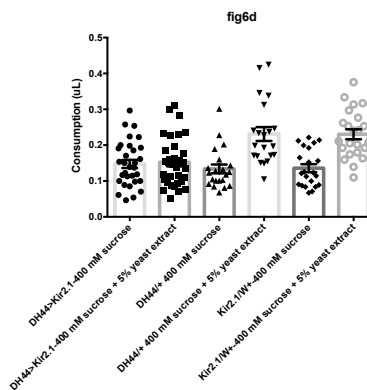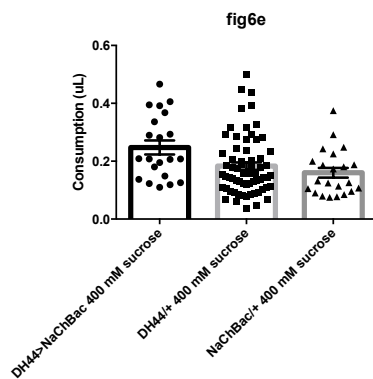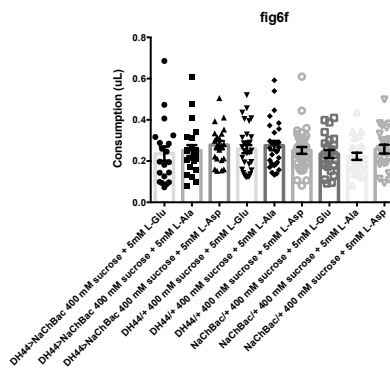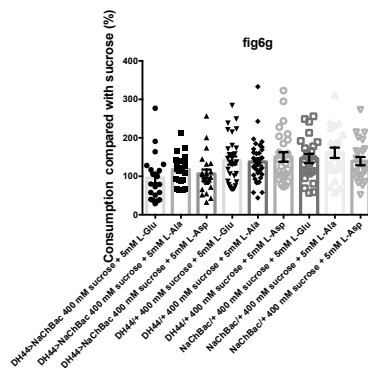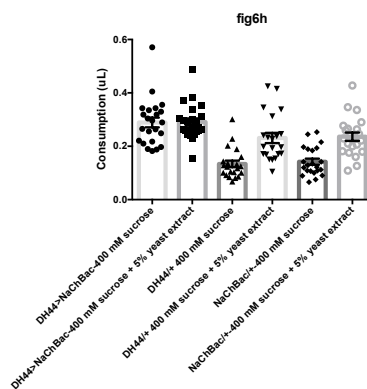

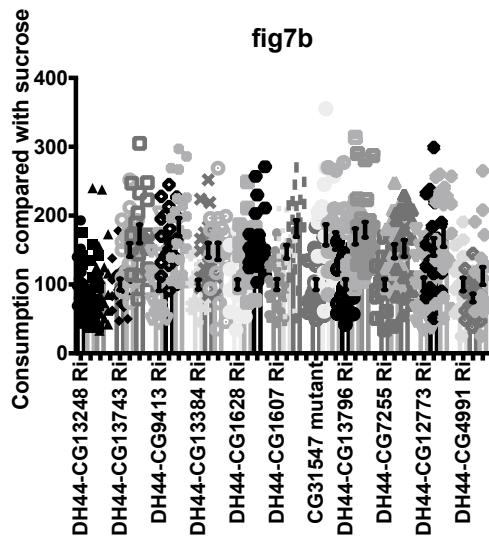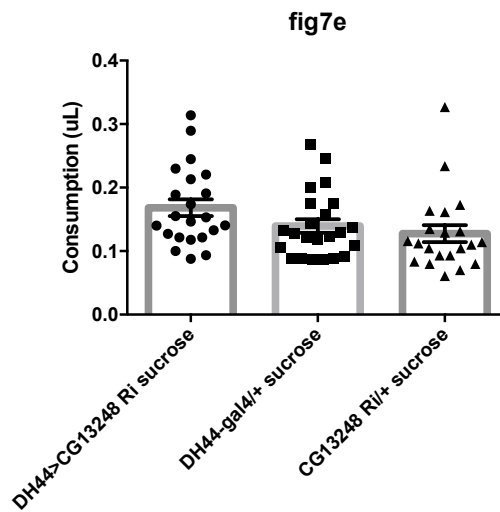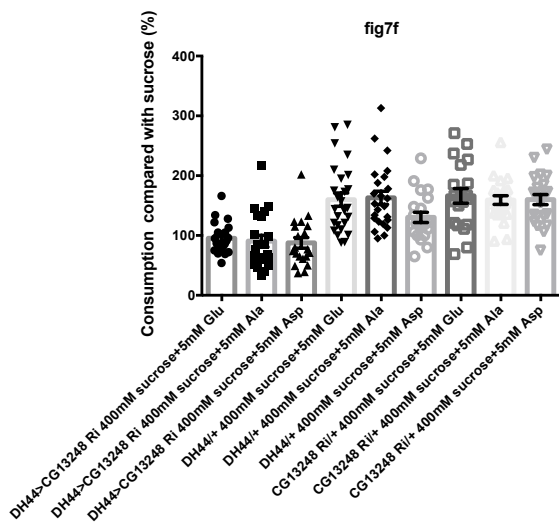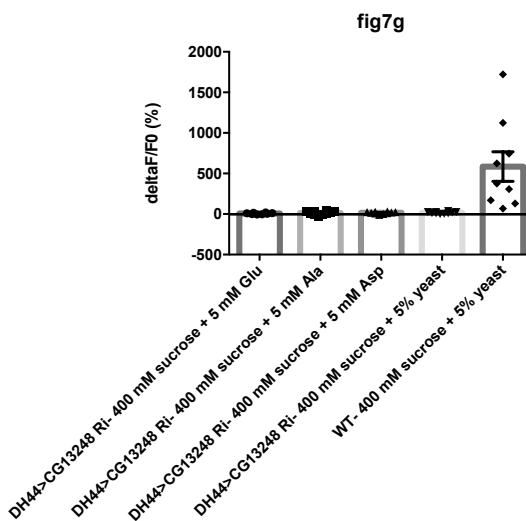

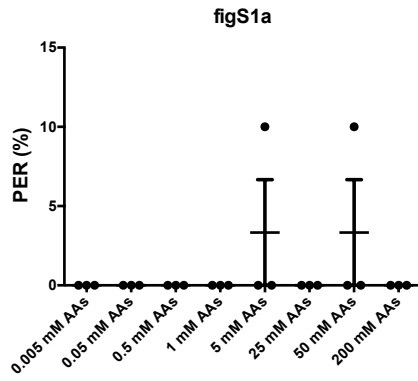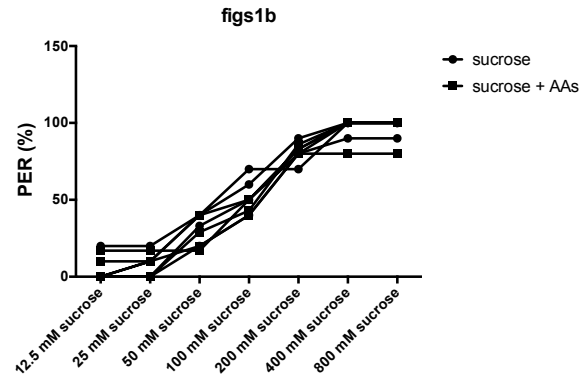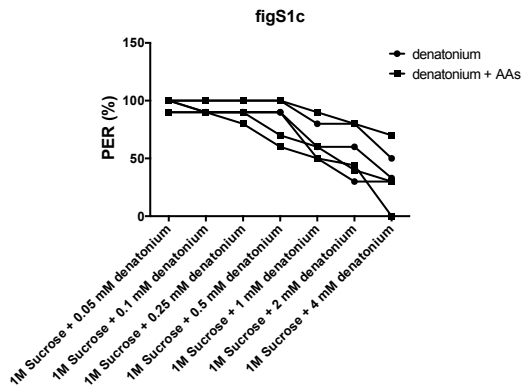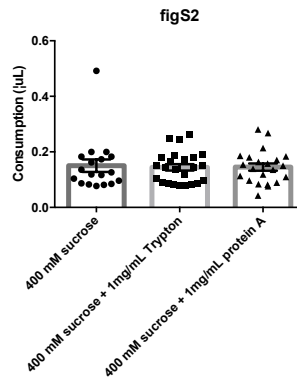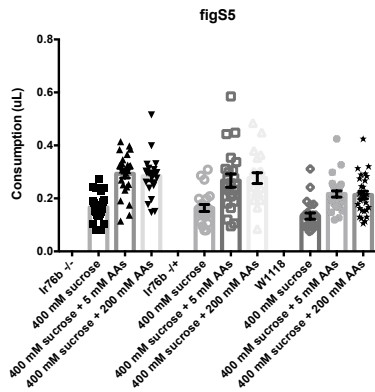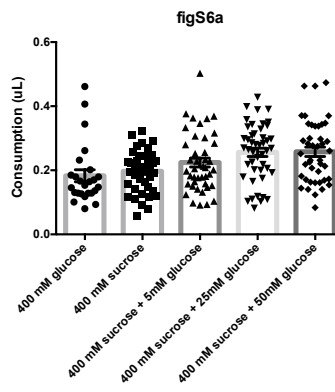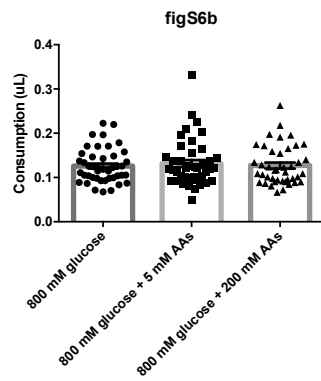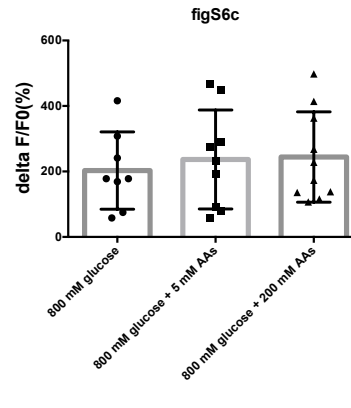

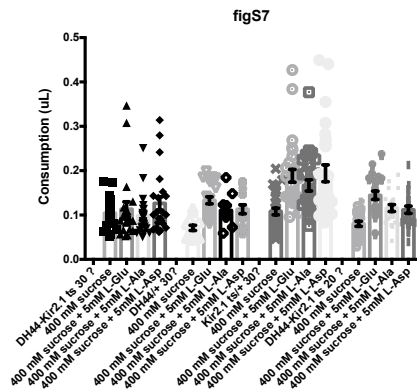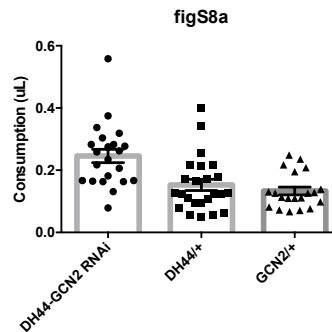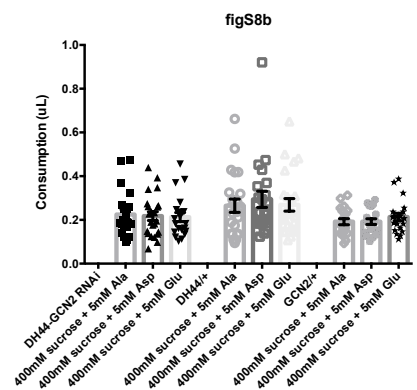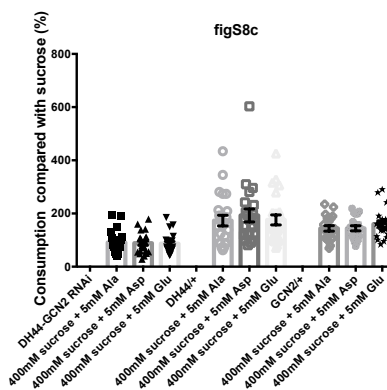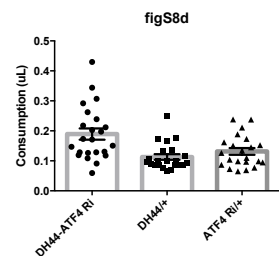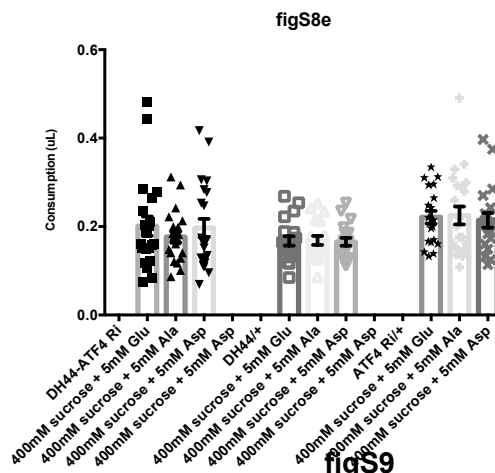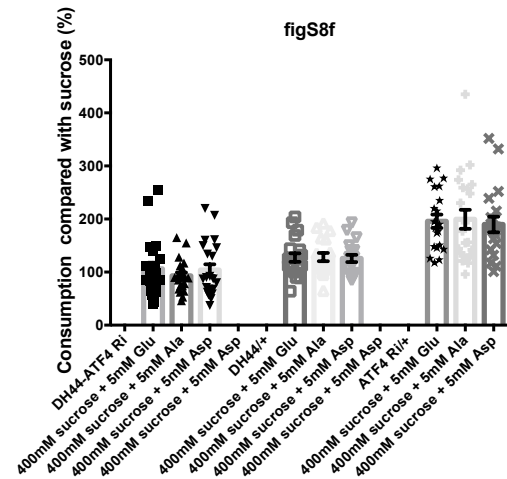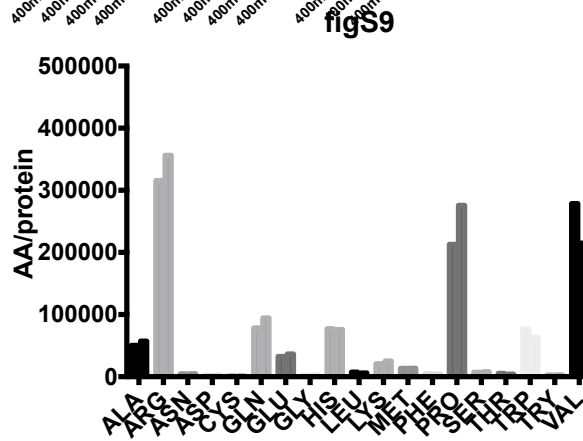

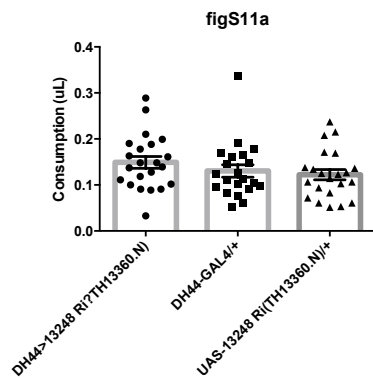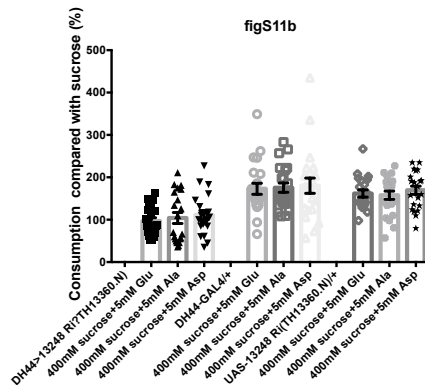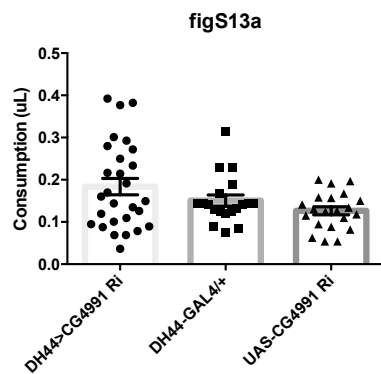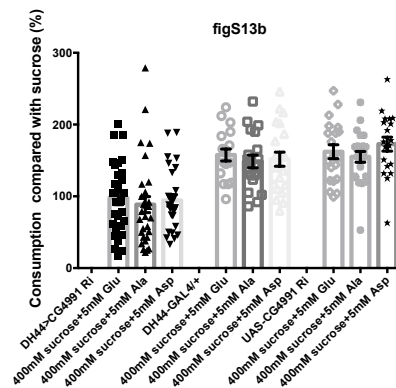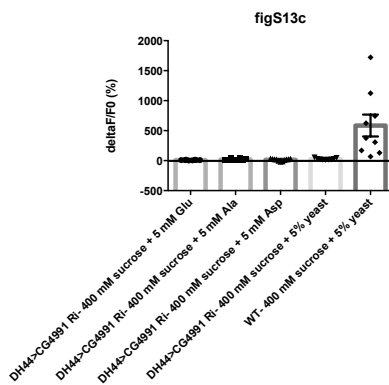

Supplement: Supplementary file 17 — Supplementary information, Data S1 [file 41422_2018_84_MOESM17_ESM.pdf]
